# Dynamics and reversibility of hepatic steatosis in male Mule duck

**DOI:** 10.3389/fphys.2026.1804237

**Published:** 2026-04-16

**Authors:** Ophélie Bernardi, Maxime Reverchon, Christelle Ramé, Eleni Zoi Glaveli, Hervé Rémignon, Pascal Froment, Joëlle Dupont

**Affiliations:** 1 Centre National de la Recherche Scientifique, Institut Français du Cheval et de l’Equitation, Institut National de la Recherche pour l’Agriculture, l’Alimentation et l’Environnement, Université de Tours, Physiologie de la Reproduction et des Comportements, UMR85, Nouzilly, France; 2 SYSAAF-Syndicat des Sélectionneurs Avicoles et Aquacoles Français, Centre INRAE Val de Loire, Nouzilly, France; 3 Toxalim, Université de Toulouse, INRAE, ENVT, EI-Purpan, Toulouse, France; 4 Ecole Nationale Supérieure Agronomique de Toulouse (INP-AgroToulouse), Auzeville-Tolosane, France

**Keywords:** duck, force-feeding, liver, reversibility, steatosis

## Abstract

**Introduction:**

This study investigated the physiological, biochemical, and molecular shifts in male Mule ducks during an assisted-feeding period and the subsequent recovery phase following the cessation of overfeeding. While assisted-feeding is known to induce significant hepatic changes, the timeline and extent of the liver's capacity to return to a basal state remain critical areas of inquiry.

**Methods:**

Male Mule ducks were subjected to a period of assisted-feeding followed by a recovery phase where they returned to an ad libitum diet. We monitored body weight and liver mass, analyzed plasma metabolic markers (including lipids and liver enzymes), and evaluated hepatic composition. Molecular analysis was conducted to assess gene expression related to lipogenesis, inflammation, and apoptosis, while antioxidant enzyme activities and hypoxia markers were measured to determine cellular stress levels.

**Results:**

Assisted-feeding significantly increased body weight, liver mass, and hepatic steatosis, accompanied by a sharp rise in plasma markers such as triglycerides, cholesterol, and liver enzymes (ALAT, LDH, ALP). At the molecular level, there was a marked upregulation of genes involved in lipogenesis (scd1, dgat2), inflammation (TNFα, IL8), and apoptosis. Furthermore, increased activities of antioxidant enzymes (SOD, Cat, GPX1) and markers of hypoxia (HIF-1α, HIF-2α) indicated significant metabolic load and cellular stress. Following the cessation of overfeeding, most physiological and biochemical parameters, including liver mass and enzyme levels, returned to control values within 20 to 29 days. Notably, while hepatic alterations were fully abolished, abdominal fat remained significantly higher in ex-force-fed ducks compared to the control group.

**Conclusion:**

The study demonstrates that hepatic steatosis induced by assisted-feeding in Mule ducks is a highly reversible process. Despite triggering significant oxidative stress, hypoxia, and inflammatory responses, the avian liver exhibits a remarkable regenerative capacity, returning to its basal physiological and molecular state within 29 days of returning to a standard diet.

## 1 Introduction

The mule duck, a sterile hybrid of male Muscovy duck and female Pekin duck, possesses a remarkable physiological capacity for hepatic steatosis, a process traditionally exploited in “foie gras” production (André et al., 2007; Chartrin et al., 2006). Foie gras is a famous specialty of the French gastronomy, which has been recognized by UNESCO as Intangible Cultural Heritage of Humanity since 2010. In birds hepatic steatosis can occur spontaneously as a consequence of energy storage (Pilo and George, 1983) prior to migration for example. During the assisted-feeding period, a carbohydrate-rich diet induces massive *de novo* lipogenesis, leading to a significant accumulation of triglycerides within the hepatocytes (Hermier et al., 1991, Hermier et al., 1999, Hermier et al., 2003; Chartrin et al., 2006). Due to high lipid deposition, inflammation, oxidative stress, cell proliferation, and apoptosis finally take place in fattening liver of assisted-fed ducks (Mozduri et al., 2021; Rémignon et al., 2018, Rémignon and Burgues, 2023, Atallah et al., 2024). Unlike mammalian non-alcoholic fatty liver disease (NAFLD), which often progresses toward irreversible inflammation and fibrosis, the hepatic steatosis developed by waterfowl is more related to a non-pathological process with a macrovesicular storage of lipids (Pilo and George, 1983).

Beyond its biological uniqueness, “foie gras” production faces growing societal concerns regarding animal welfare. The practice of assisted-feeding is increasingly debated, leading to strict regulations and, in some regions, bans on production. Some studies sometimes contradictory, have already reported an impact of the assisted-feeding procedure itself on mule ducks health and welfare (Guémené et al., 2001, Guémené et al., 2006; Flament et al., 2012; Rochlitz and Broom, 2017). In this context, demonstrating the reversibility of hepatic steatosis is a major scientific and ethical challenge. This study aims to report that the liver can return to its baseline limits in animals and to address concerns about the potentially pathological nature of the process. A defining characteristic of this phenomenon is indeed its potential for recovery. In a production context, studies in geese and Mule duck have shown that if overfeeding is ceased, the liver weight and lipid content return to basal levels within a few weeks (Babilé et al., 1998; Bénard and Labie, 1998). However, the increase in the final liver weight is always associated with a hypoxic response and the massive influx of lipids and the subsequent increase in metabolic activity during overfeeding generate reactive oxygen species (ROS), potentially leading to oxidative stress (Lo et al., 2020). In mammals, this stress triggers pro-inflammatory pathways and apoptosis (programmed cell death), driving the transition from simple steatosis to steatohepatitis (NASH). In contrast, ducks appear to maintain hepatic integrity through an efficient antioxidant system and a controlled inflammatory response. However, apoptosis occurs belatedly when an accumulation of large quantities of lipids in liver is observed (Rémignon et al., 2018). The role of enzymatic defenses, such as superoxide dismutase (SOD) and catalase (CAT), as well as the balance of the GSH/GSSG ratio, is crucial in neutralizing ROS and maintaining the cellular redox status (Pioche et al., 2020). However, the hypoxic response, the oxidative stress and the apoptotic pathways have never been investigated following the cessation of the assisted-feeding in duck.

The objective of this study was to investigate the reversibility of hepatic steatosis in mule ducks following a period of overfeeding. By monitoring morphological changes, plasma parameters, lipid metabolism markers, apoptosis, and the activity of key antioxidant systems (SOD, CAT, and GSH/GSSG), we seek to characterize the efficiency of the post-overfeeding recovery phase. This research aims to provide a better understanding of the animal’s resilience, contributing to both the biomedical field and the societal dialogue regarding waterfowl production.

## 2 Materials and methods

### 2.1 Ethical issues

All experimental procedures were carried out in accordance with the Ethics Committee (approval no. APAFIS#25453–202004302222437 v4) and the French National Guidelines for the Care of Animals for Research Purposes.

### 2.2 Animals and experimental procedures

The ducks used were male mule ducks (Cairina moschata × Anas platyrhynchos, n=108, **Supplementary Figure 1**). They were reared in a 30 m² building from one day of age; from 4 weeks of age, they were given outdoor access (1.04 ducks/m²). They were raised on wood chip litter under natural lighting, with water provided via nipple drinkers at the Experimental Station for Waterfowl Breeding (INRAE Artiguères, France). The ducks were fed *ad libitum* with a growing diet [17.5% crude protein (CP), 2,850 kcal/kg metabolizable energy (ME)] from hatching until 8 weeks of age. A finishing diet (15.5% CP, 2,800 kcal/kg ME) was then introduced, distributed via hourly rationing (1 h/day) from weeks 8 to 9, followed by quantitative rationing from weeks 9 to 12 to prepare for overfeeding (Arroyo et al., 2018).

At 12 weeks of age (day 84), the ducks were assisted-fed for 11 days (22 meals, 2 meals per day). The diet consisted of a mash (53% dry matter (DM), Palma from Maïsadour, Haut-Mauco, France) composed of 98% corn and 2% premix (3,230 kcal/kg ME; 7.2% CP, 3.2% fat, 2% crude fiber, 2.1% ash, 0.23% lysine, 0.15% methionine, 0.12% calcium, 0.25% phosphorus, and 0.1% sodium) mixed with 47% water, following the intake curve shown in **Supplementary Figure 2**. Following the end of the overfeeding period (day 96), the remaining animals were fed *ad libitum* until day 125. At specific time points—during the overfeeding period (days 84, 89, 96) and the post-overfeeding period (days 104, 117, 125)—14 animals were slaughtered according to the standardized slaughter operations (electronarcosis, bleeding, scalding, and plucking). During the overfeeding period, slaughter occurred two hours after the final meal. Additionally, a control group of eight *ad libitum* fed animals (never overfed) was slaughtered on days 104, 117, and 125. Blood samples were collected in heparinized tubes, and plasma was separated by centrifugation (3,000 × g for 10 min at 4 °C) and stored at −20 °C. After dissection, the liver, breast muscle (pectoralis major), and abdominal fat were weighed and sampled, then flash-frozen in liquid nitrogen and stored at −80 °C for subsequent molecular analysis.

### 2.3 Plasma biochemical assays

Plasma total cholesterol, bilirubin, triglyceride, ALAT, LDH, ALP, creatinine and urea levels were quantified by colorimetric enzymatic methods using kits provided by BioMérieux (Marcy-l’Etoile, France), according to the manufacturer’s recommendations.

### 2.4 Biochemical composition of livers

The dry matter content of livers was determined by drying the ground liver in an oven at 105 °C for 24 h. The total lipid content was measured according to Folch et al. (1957) after extraction with chloroform:methanol (2:1). Total protein content was determined according to the manufacturer’s procedure (*Pierce™ BCA protein assay kit, 23227, ThermoFisher, Fisher Scientific, Strasbourg, France*)) after extraction with phosphate-buffered saline. The hydroxyproline (OH-Pro) content was determined according to the methodology described by Woessner (1961) on the delipidated dry residue obtained after the extraction of total lipids.

### 2.5 Oxidative status

The ratio of reduced glutathione to oxidized glutathione (GSH/GSSG ratio) was measured following the manufacturer’s protocol (Cat. No. ab239709, Abcam, Cambridge, UK). Results are expressed in µmol/mg of protein. The activities of superoxide dismutase (SOD; Cat. No. 19160, Sigma-Aldrich, St. Louis, MO, USA) and catalase (CAT; Cat. No. KB03012, BioQuoChem, Llanera, Asturias, Spain) were determined according to the manufacturers’ instructions. Results are expressed in U/mg of protein.

### 2.6 Caspases activities assays

The activities of caspase-3/7 (Cat. No. ab39401), caspase-8 (Cat. No. ab39700), and caspase-9 (Cat. N°. Ab65607) enzymes were determined using the assay kits from Abcam (Cambridge, UK) according to the manufacturer’s instructions. For each enzyme, the total activity of a pooled liver extract, consisting of all available samples, was measured alongside the individual samples. The activity of the pooled extract was then arbitrarily set to 100, and the activity of each individual sample was expressed as a percentage of this value, with enzyme activities reported in arbitrary units.

### 2.7 ELISA assays

The levels of Hypoxia Inducible Factor 1 Alpha (HIF1α, catalogue: # MBS065720), Hypoxia Inducible Factor 2 Alpha (HIF2α, catalogue:# MBS9365131), Glutathione Peroxidase (GPX, catalogue: #MBS061664) and NQO1 (Nicotinamide Adenine Dinucleotide (Phosphate) Hydrogen Dehydrogenase (Quinone) 1, catalogue: #MBS061664) were quantified by ELISA tests using MyBioSource (MBS, San Diego, CA, USA) assay kits in accordance with the manufacturer’s instructions. The results are expressed as pg or ng/mg protein. The intra and inter-assay coefficients of variation (CV) averaged <10%.

### 2.8 Gene expression analysis

Total RNAs were extracted from duck livers using the TRIzol RNA Isolation Reagents and an Ultraturax instrument for grinding, according to the manufacturer’s recommendations (Invitrogen, by Life Technologies, Villebon sur Yvette, France). The purity and the concentrations of the obtained RNAs were checked via their A260/A280 ratios using a Nanodrop machine. cDNAs were obtained by reverse transcription of 2 µg of the total RNAs in 20 µL of a mix containing 0.5 mM of each deoxyribonucleotide triphosphate (dATP, dTTP, dGTP, dCTP), 2 M of RT Buffer, 15 μg/μL of oligodT, 0.125 U of ribonuclease inhibitor, and 0.05 U of Moloney murine leukemia virus reverse transcriptase (MMLV) were placed 1 hour at 37 °C. Quantitative PCR was performed using a mix of 3 µL of cDNA and 8 µL of SYBR Green Supermix 1X Reagent (Bio-Rad, Marnes-la-Coquette, France) with 250 nM of specific primers (Invitrogen by Life Technologies, Villebon-sur-Yvette, France) given **Table 1**. Samples were set up in duplicate in a 384 wells plate and a MyiQ Cycle Device (Bio-Rad, Marnes-la-Coquette, France) was used to apply the following procedure: incubation (2 min at 50 °C), denaturation (10 min at 95 °C), 40 PCR cycles (30 s at 95 °C, 30 s at 60 °C, 30 s at 72 °C). Relative expressions of genes were related to the expression of three reference genes (GAPDH (Glyceraldehyde-3-phosphate dehydrogenase), ACTB (Actin B) and EEF1A (Eukaryotic Elongation Factor 1 alpha)) geometric mean. For each target gene, expression was calculated according to primer efficiency (E) and quantification cycle (Cq), where expression = E-Cq. Then, relative expression of the target gene to the three reference genes was analyzed.

**Table 1:** Oligonucleotide sequences of primers used for qRT-PCR.

	Forward	Reverse	references
Lipogenesis			
*acsl1* (Acyl CoA synthetase long-chain 1)	GGCTGGCTTCATACAGGAGA	CTCTTTTCTTGGCCCATTTG	*Pioche* et al.*, 2020*
*scd1* (Steraoyl-CoA 9 desaturase)	ATGCCTGCGCACTTGCTG	ACGGTGGTGGTGCTGGAA	*Pioche* et al.*, 2020*; *Atallah* et al.*, 2024*
*dgat2* (Diacyglycérol O-acyltranferase 2)	CTGGGCTATATGGAGGTACTTCAG	TGGTCAGCAGATTGTGGGTTT	*Pioche* et al.*, 2020*; *Atallah* et al.*, 2024*
β-oxidation			
*cpt1a* (Carnitine palmitoyl transferase 1)	TGGACAGACAAGAAATCAAGCCA	CGGAACAGTTGATCCCATCAGAA	*Pioche* et al.*, 2020*
*Acad11* (Acyl CoA dehydrogenase 11)	TGGTTGTACCTCGAGCTGTG	CATCCACATGAGAGGGCTTT	*Pioche* et al.*, 2020*
Lipoprotein formation			
*Apob* (Apolipoprotein B)	ACCTGCCTGTTATCACCATTCC	TGTATTTGATCCGGCCTTCACTT	*Pioche* et al.*, 2020*; *Atallah* et al.*, 2024*
*cept1* (Cholesteryl ester transfer protein 1)	CTGCTGTGCAGCTCTTTGAA	CGCAGTATCGAAGCAAATCA	*Pioche* et al.*, 2020*
*plin2* (Perilipin 2)	CGAGTATGCCAGAAAGAACATGAATAG	TCTTCCATTCTACCCAGGATTGATAC	*Pioche* et al.*, 2020*; *Atallah* et al.*, 2024*
Fatty acid maturation and transport			
*fabp4 (*Fatty acid binding protein 4)	AATGGCTCACTGAAGCAGGT	TGGCTTCTTCATGCCTTTTC	*Atallah* et al.*, 2024*
*fat/cd36 (Fatty acid translocase/cluster of differentiation 36)*	AGTTTGCCAAAAGGCTTCAA	CGAGGAACACCACAGAACCT	*Pioche* et al.*, 2020*
Lipoprotein receptors			
*Vldlr* (Very low-density lipoprotein receptor)	CGTAACTGGCAGCACAAGAA	TGCTGATCCAGTGCTCAAAC	*Pioche* et al.*, 2020*
*Ldlr* (Low-density lipoprotein receptor)	TGTGGCCTTCAGAAAGCTCG	ATCTCGTGCTGCATGTAGGG	*Pioche* et al.*, 2020*
Cholesterol synthesis and esterification			
*Hmgcr* (Hydroxymethylglutaryl-CoA reductase)	CATTTTGCTCGTGTTCTGGA	ATCCATACTGGCCATTCGAG	*Pioche* et al.*, 2020*
*soat1* (Sterol o-acyltransferase 1)	CTCTTCTGCCTGTTTATGTG	GACGGTCGTTAAGAATGAAG	*Pioche* et al.*, 2020*; *Atallah* et al.*, 2024*
Inflammation			
*TNF𝛼* (Tumor Necrosis Factor alpha)	GCCACTGATGTCTTCAATTCCAAA	ATCTTCTTCTGGGCCTGAATGG	*Atallah* et al.*, 2024*
*IL8*	TGGAGAGAACCTCTGCCTCTATT	AGAAGGCATCACATTCCAGCTC	*Atallah* et al.*, 2024*
Apoptosis			
*Caspase-3*	GCAGACAGTGGACCAGATGA	CTTGCATGTTCCTTCAGCAC	*Pioche* et al.*, 2020*
*Caspase-8*	GGAAAGCAGTGCCAGAACTC	TAAAATGAAGGGTGCCGAAG	*Pioche* et al.*, 2020*
*Caspase-9*	CACCCGAAGATGAAACTTGC	CCATGATCCGCTCGACTTAT	*Pioche* et al.*, 2020*
*Bax*	GAGAGCGGCGACAAGGATTACATC	CTCGTTCATGCCCAGGATCATCAG	*Hu* et al.*, 2024*
*Bcl-2*	GAGTTCGGCGGCGTCATGTG	CCATACAACTCCACGAAGGCATCC	*Hu* et al.*, 2024*
Cellular Stress			
*Cyp2e1* (Cytochrome P450 2E1)	GCCAGATGCCCTACACAGAT	GGTACCGTTTGGATTCAGGA	*Pioche* et al.*, 2020*
*Hspb1* (Heat shock protein 1)	ACTGGAAGGCGAGAACAAGA	TCGGTAATTCCAAGGGACTG	*Pioche* et al.*, 2020*
References gene			
*BACTIN*	CCAGCCATCTTTCTTGGGTA	ATGCCTGGGTACATTGTGGT	*Tavernier* et al.*, 2020*
*EEF1A*	AGCAGACTTTGTGACCTTGCC	TGACATGAGACAGACGGTTGC	*Bernardi* et al.*, 2023*
*GAPDH*	CAGAGGACCAGGTTGTCTCC	CACCACACGGTTGCTGTATC	*Tavernier* et al.*, 2020*

### 2.9 Statistical analysis

Statistical analysis was carried out by GraphPad Prism 8.0.2 software, and statistical significance was defined as *P* < 0.05. Data are presented as mean ± standard error of the mean (SEM). Normal distribution of the data was assessed by the Kolmogorov–Smirnov test. For normally distributed data, Student’s t–test was used for two-group comparisons, and one-way ANOVA followed by the Tukey test was used for multiple-group comparisons. Different letters indicate significant differences (*P* < 0.05).

## 3 Results

### 3.1 Body weight, tissue weights, and plasma biochemistry in male Mule ducks during and following the cessation of assisted-feeding

**Figure 1:**
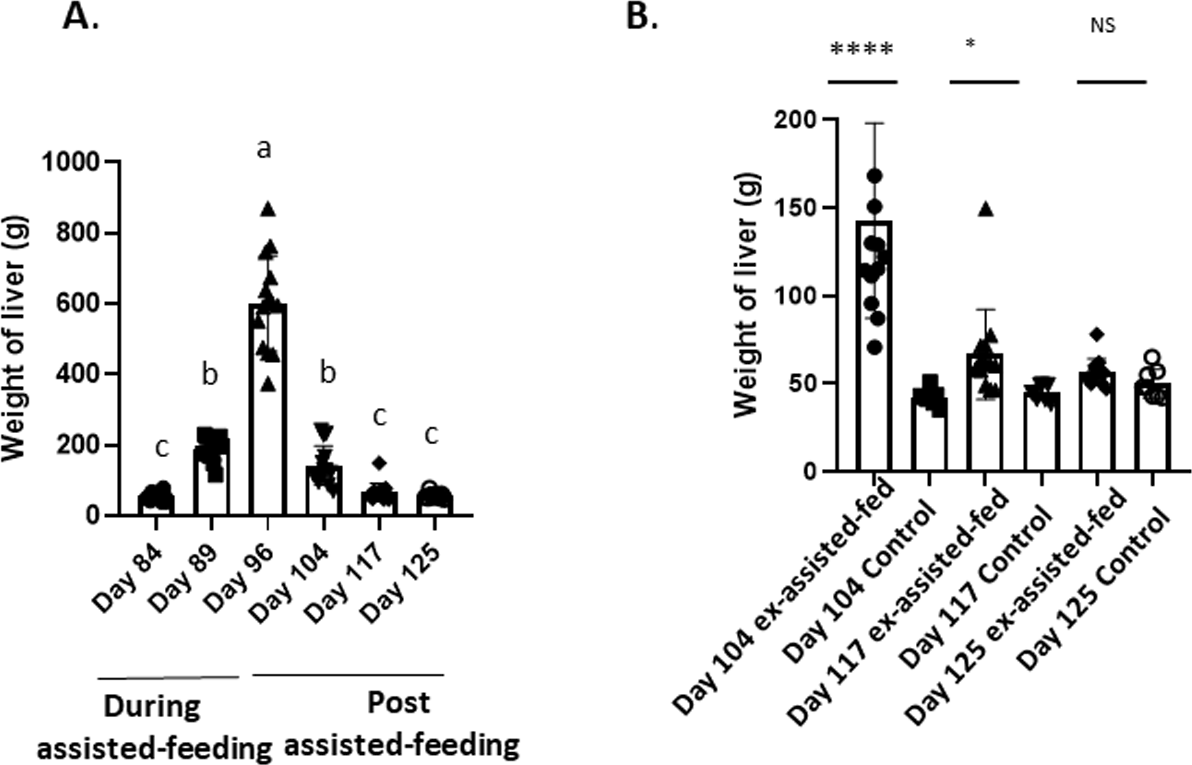
Changes in liver weight during and the post assisted-feeding period. **(A)** Liver weight (g) at specific time points (n = 14) during the assisted-feeding period (days 84, 89, 96) and the post-overfeeding period (days 104, 117, 125). Data are presented as mean ± SEM. Different letters (a-c) denote significant differences between sampling points (p < 0.05). Groups sharing the same letter are not significantly different. **(B)** Comparison of liver weight (g) at specific time points (days 104, 117, 125) between previously assisted-fed ducks (n = 14) and *ad libitum* control ducks (n = 8). Significant differences are indicated by *p < 0.05 and ****p < 0.0001. NS: not significant.

The assisted-feeding and the transition assisted-feeding/*ad libitum* program took place without any noticeable events (mortality <1%, only 2 animals were dead). To investigate the mechanisms underlying the development of hepatic steatosis and the possible reversibility, zootechnical data (tissue weights) and plasma parameters were first analyzed during and after overfeeding (n=14 to each sampling points, **Table 2**). In order to confirm a potential reversibility of the alterations observed during the assisted-feeding we also compared animals 8, 21 and 29 days after the over-feeding period with control *ad libitum* feeding animals (control, n=8 to each sampling points) that have never been assisted-fed (**Table 3**). As shown in **Table 2**, a significant increase during the assisted-feeding and then a reduction after the arrest of the food practice in body weight (P<0.0001), liver (P<0.0001, **Figure 1**), and abdominal fat (P<0.0001) was observed. The return to control weight as determined in *ad libitum* animals was reached at 20 and 29 days after the assisted-feeding arrest for the body weight and liver mass (, respectively whereas the abdominal fat amount was still significantly higher in ex-assisted-fed animals compared to the control at these two periods. At the opposite the breast mass (without subcutaneous fat) remained stable during the assisted-feeding whereas it slightly decreased 21 days after the assisted-feeding arrest.

**Table 2:** Changes in body weight, liver mass, breast mass, and abdominal fat content in Mule ducks during overfeeding from day 84 to day 96, and following the cessation of overfeeding under *ad libitum* feeding conditions on days 104, 117, and 125.

During over-feeding				After over-feeding			

Age of animals (days)	**84**	**89**	**96**	**104**	**117**	**125**	***P***
Time from the beginning and after the over-feeding period (day)	**0**	**5**	**12**	**8**	**21**	**29**	
	n=14	n=14	n=14	n=14	n=14	n=14	
Feeding level	**Control**	**Overfed**	**Overfed**	***Ad-libitum***	***Ad-libitum***	***Ad-libitum***	
Body weight (g)	4134 ± 117^a^	4904 ± 98**^b^**	6044 ± 127**^c^**	5008 ± 88**^b^**	4831 ± 183**^b^**	4539 ± 150 a**^,b^**	**0.0001**
Liver mass (g)	55 ± 3**^a^**	190 ± 9**^b^**	600 ± 37**^c^**	143 ± 15**^b^**	67 ± 7**^a^**	57 ± 2**^a^**	**0.0001**
Variation of liver mass (%/control Day 84)	100 ± 6**^a^**	345 ± 15**^b^**	1090 ± 65**^c^**	259 ± 27**^b^**	121 ± 12**^a^**	103 ± 4**^a^**	**0.0001**
Breast mass (g)	314 ± 11**^a^**	313 ± 8 a**^,b^**	329 ± 11 a**^,b^**	295 ± 6 a**^,b^**	277 ± 12**^b^**	320 ± 18 a**^,b^**	**0.02**
Abdominal fat (g)	29 ± 4**^a^**	66 ± 6**^b^**	145 ± 8**^c^**	140± 5**^c^**	91 ± 5**^d^**	70 ± 5**^b^**	**0.0001**

Values are expressed as means ± SEM, n=14 at each specific time point. One-way ANOVA was performed followed by the Tukey test. Letters a,b, c and d denotes significant differences between sampling points.

The variation in liver mass relative to the control at day 84 is also shown.

The P values significant are in bold.

**Table 3:** Changes in body weight, liver mass, breast mass, and abdominal fat in Mule ducks following the cessation of overfeeding, in comparison with control animals that were never overfed.

Age of animals (days)	104			117			125		
**Time from the cessation of the over-feeding period (day)**	**8**			**21**			**29**		
	n=14	n=8		n=14	n=8		n=14	n=8	
**Feeding level**	**Ex-Overfed** ***Ad-libitum***	**Control** ***Ad-libitum***	***P***	**Ex-Overfed** ***Ad-libitum***	**Control** ***Ad-libitum***	***P***	**Ex-Overfed** ***Ad-libitum***	**Control** ***Ad-libitum***	***P***
**Body weight (g)**	5008 ± 88	4020 ± 139	**0.0001**	4831± 183	4531 ± 79	0.23	4539 ± 150	4505 ± 176	0.97
**Liver mass (g)**	143 ± 15	42 ± 2	**0.0001**	67 ± 7	45 ± 2	**0.02**	57 ± 2	46 ± 3	0.06
**Breast mass (g)**	295 ± 6	313 ± 10	0.12	277 ± 12	355 ± 11	**0.0002**	320 ± 18	364 ± 21	0.12
**Abdominal fat (g)**	140± 5	13 ± 2	**0.0001**	91 ± 5	34 ± 4	**0.0001**	70 ± 5	32 ± 7	**0.0001**

Data are presented as means ± SEM, with n = 14 for the Ex-Overfed group and n = 8 for the Control group. Comparisons between groups were performed using a Student’s t-test.

The P values significant are in bold.

In addition, as shown in **Table 4**, analyses of plasma parameters (total cholesterol, ALAT, LDH, ALP, bilirubin, triglycerides and creatinine) showed a significant increase during the assisted-feeding period and a significant decrease after the arrest of this latter period. Indeed, plasma total cholesterol and ALP concentrations were restored 8 days after the arrest of the assisted-feeding, LDH, bilirubin and creatinine 20 days and triglycerides and ALAT 29 days after the arrest of over-feeding (**Table 5**). Only the urea plasma concentrations were not significantly affected by the assisted-feeding (**Table 4**).

**Table 4:** Changes in lipid content, dry matter, protein levels, and hydroxyproline (OH-Pro) in Mule duck livers during overfeeding period (days 84–96) and following the cessation of overfeeding under *ad libitum* conditions.

During over-feeding				After over-feeding			

Age of animals (days)	**84**	**89**	**96**	**104**	**117**	**125**	***P***
Time from the beginning and after the over-feeding period (day)	**0**	**5**	**12**	**8**	**21**	**29**	
	n=14	n=14	n=14	n=14	n=14	n=14	
Feeding level	**Control**	**Overfed**	**Overfed**	***Ad-libitum***	***Ad-libitum***	***Ad-libitum***	
Lipids (% raw tissue)	5.6 ± 0.3**^a^**	25.1 ± 0.5**^b^**	56.9 ± 1.4**^c^**	27.6 ± 1.0**^b^**	19.7 ± 1.3**^a^**	5.6 ± 0.4**^a^**	**0.0001**
Dry matter (% raw tissue)	33.0 ± 0.5**^a^**	44.3 ± 0.8**^b^**	74.8 ± 0.9**^c^**	48.4 ± 2.1**^b^**	36.8 ± 0.7**^a^**	33.2 ± 0.7**^a^**	**0.0001**
Proteins (% raw tissue)	12.8 ± 0.4**^a^**	4.4 ± 0.3**^b^**	2.9 ± 0.2**^c^**	4.8 ± 0.3**^b^**	12.1 ± 0.3**^a^**	12.6 ± 0.3**^a^**	**0.0001**
OH-Pro (mg/g delipidated and dried tissue)	0.43 ± 0.02 a**^,b,d^**	0.46 ± 0.02 a**^,d^**	0.39 ± 0.01**^b,c^**	0.38 ± 0.01**^c^**	0.43 ± 0.01 a**^,b,d^**	0.47 ± 0.005**^d^**	**0.0001**

Values are expressed as means ± SEM, n=14 at each specific time point. One-way ANOVA was performed followed by the Tukey test. Letters a,b, c and d denotes significant differences between sampling points. OH-Pro: hydroxyproline.

The P values significant are in bold.

**Table 5:** Changes in lipid content, dry matter, protein levels, and hydroxyproline (OH-Pro) in Mule duck livers following the cessation of overfeeding, in comparison with control animals that were never overfed.

Age of animals (days)	104			117			125		
Time from the cessation of the over-feeding period (day)	**8**			**21**			**29**		
	n=14	n=8		n=14	n=8		n=14	n=8	
Feeding level	**Ex-Overfed** ***Ad-libitum***	**Control** ***Ad-libitum***	***P***	**Ex-Overfed** ***Ad-libitum***	**Control** ***Ad-libitum***	***P***	**Ex-Overfed** ***Ad-libitum***	**Control** ***Ad-libitum***	***P***
Lipids (% raw tissue)	27.6 ± 1.0	5.1 ± 0.5	**0.0001**	19.7 ± 1.3	4.9 ± 0.5	**0.0001**	5.6 ± 0.4	5.0 ± 0.4	0.34
Dry matter (% raw tissue)	48.4 ± 2.1	32.9 ± 0.7	**0.0001**	36.8 ± 0.7	34.2 ± 0.4	**0.02**	33.2 ± 0.7	32.5 ± 0.6	0.50
Proteins (% raw tissue)	4.8 ± 0.3	12.8 ± 0.4	**0.0001**	12.1 ± 0.3	12.5 ± 0.5	0.5	12.6 ± 0.3	13.1 ± 0.4	0.40
OH-Pro (mg/g delipidated and dried tissue)	0.38 ± 0.01	0.47 ± 0.01	**0.0001**	0.43 ± 0.01	ND	ND	0.47 ± 0.005	0.47 ± 0.01	0.65

Data are presented as means ± SEM, with n = 14 for the Ex-Overfed group and n = 8 for the Control group. Comparisons between groups were performed using a Student’s t-test. OH-Pro: hydroxyproline. ND: not determined.

The P values significant are in bold.

### 3.2 Liver composition in male Mule ducks during and after assisted-feeding

As shown in **Table 6**, the dry matter and lipid contents in liver strongly increased during the assisted-feeding period (from day 84 to day 96) while the protein one decreased. These effects were totally abolished 20 days (for protein contents) and 29 days (lipid and dry matter content) after the arrest of assisted-feeding (**Table 7**). The total OH-Pro content in liver remained similar during the overfeeding period (**Table 6**).

**Table 6:** Changes in plasma parameters in Mule ducks during overfeeding (days 84–96) and after the cessation of overfeeding under *ad libitum* conditions.

During over-feeding				After over-feeding in *ad libitum* condition			
Age of animals (days)	**84**	**89**	**96**	**104**	**117**	**125**	***P***
Time from the beginning and after the over-feeding period (day)	**0**	**5**	**12**	**8**	**21**	**29**	
	n=14	n=14	n=14	n=14	n=14	n=14	
Feeding level	**Control**	**Overfed**	**Overfed**	***Ad-libitum***	***Ad-libitum***	***Ad-libitum***	
Cholesterol (mg/L)	4.4 ± 0.1^a^	4.8 ± 0.1^a^	5.4± 0.2 ^b^	4.4 ± 0.1 a	4.1 ± 0.1 ^a^	4.2 ± 0.1 a	**0.0001**
ALAT (U/L)	23.4± 0.4^a^	30.8± 0.7 ^b^	43.3± 0.7 ^c^	28.4± 0.6 ^d^	24.1± 0.2 ^a^	23.2± 0.2 ^a^	**0.0001**
LDH (U/L)	3026.0± 23.2^a^	3626.0± 77.4 ^b^	4801.0± 72.3 ^c^	3861.0± 80.6 ^b^	3060.0± 27.6 ^a^	3049± 22.1^a^	**0.0001**
ALP (U/L)	142.8± 1.8 ^a^	139.8± 1.7 ^a^	129.4± 0.9 ^b^	136.9± 2.5 a^,b^	140.9± 1.8 ^a^	147.5± 2.2 ^a^	**0.0001**
Bilirubin	4.7± 0.3 ^a^	8.2± 0.3 ^b^	14.9± 0.3 ^c^	8.1± 0.4 ^b^	4.0± 0.1 ^a^	4.1± 0.2 ^a^	**0.0001**
Triglycerides	1.1± 0.04 ^a^	2.6± 0.06 ^b^	4.0± 0.06 ^c^	2.5± 0.08 ^b^	1.4± 0.09 ^a^	1.2± 0.07^a^	**0.0001**
Creatinine (μmol/L)	12.2± 0.7^a^	14.6± 0.7 a^,c^	23.1± 1.1 ^b^	15.6± 0.5 ^c^	11.9± 0.3 ^a^	11.9± 0.2^a^	**0.0001**
Urea (mmol/L)	0.6± 0.01	0.6± 0.01	0.7± 0.02	0.6± 0.02	0.6± 0.01	0.6± 0.01	0.08

Values are expressed as means ± SEM, n=14 at each time point. ALAT: Alanine aminotransferase; LDH: Lactate dehydrogenase; ALP: alkaline phosphatase. One-way ANOVA was performed followed by the Tukey test. Letters a,b, c denotes significant differences between sampling points.

The P values significant are in bold.

**Table 7:** Changes in plasma parameters in Mule ducks following the cessation of overfeeding, in comparison with control animals that were never overfed.

Age of animals (days)	104			117			125		
Time from the cessation of the over-feeding period (day)	**8**			**21**			**29**		
	**n=14**	**n=8**		**n=14**	**n=8**		**n=14**	**n=8**	
Feeding level	**Ex-Overfed** ***Ad-libitum***	**Control** ***Ad-libitum***	***P***	**Ex-Overfed** ***Ad-libitum***	**Control** ***Ad-libitum***	***P***	**Ex-Overfed** ***Ad-libitum***	**Control** ***Ad-libitum***	***P***
Cholesterol (mg/L)	4.4 ± 0.1	4.3 ± 0.1	0.5	4.1 ± 0.1	4.2 ± 0.1	0.6	4.2 ± 0.1	4.2 ± 0.1	0.90
ALAT (U/L)	28.4± 0.6	22.6± 0.6	**0.0001**	24.1± 0.2	22.6± 0.2	**0.0001**	23.2± 0.2	22.5± 0.2	0.06
LDH (U/L)	3861.0± 80.6	3013.0± 24.9	**0.0001**	3060.0± 27.6	2983.0± 19.2	0.06	3049± 22.1	3019± 12.6	0.35
ALP (U/L)	136.9± 2.5	142.3± 2.7	0.18	140.9± 1.8	142.4± 2.0	0.60	147.5± 2.2	141.9± 2.6	0.12
Bilirubin	8.1± 0.4	4.4± 0.4	**0.0001**	4.0± 0.1	4.2± 0.4	0.53	4.1± 0.2	4.3± 0.4	0.52
Triglycerides	2.5± 0.08	1.0± 0.06	**0.0001**	1.4± 0.09	1.1± 0.04	**0.02**	1.2± 0.07	1.02± 0.04	0.06
Creatinine (μmol/L)	15.6± 0.5	12.0± 0.3	**0.0001**	11.9± 0.3	11.9± 0.1	0.9	11.9± 0.2	11.9± 0.1	0.90
Urea (mmol/L)	0.6± 0.02	0.6± 0.01	0.2	0.6± 0.01	0.6± 0.01	0.8	0.6± 0.01	0.6± 0.01	0.17

Values are expressed as means ± SEM, n=14 at each time point for the ex-overfed animals and n=8 for animals that have never been assisted-fed. ALAT: Alanine aminotransferase; LDH: Lactate dehydrogenase; ALP: alkaline phosphatase. The Ex-Overfed and control conditions were compared by a Student T Test.

The P values significant are in bold.

### 3.3 Oxidative stress markers in the liver of male Mule ducks during and after assisted-feeding

**Figure 2:**
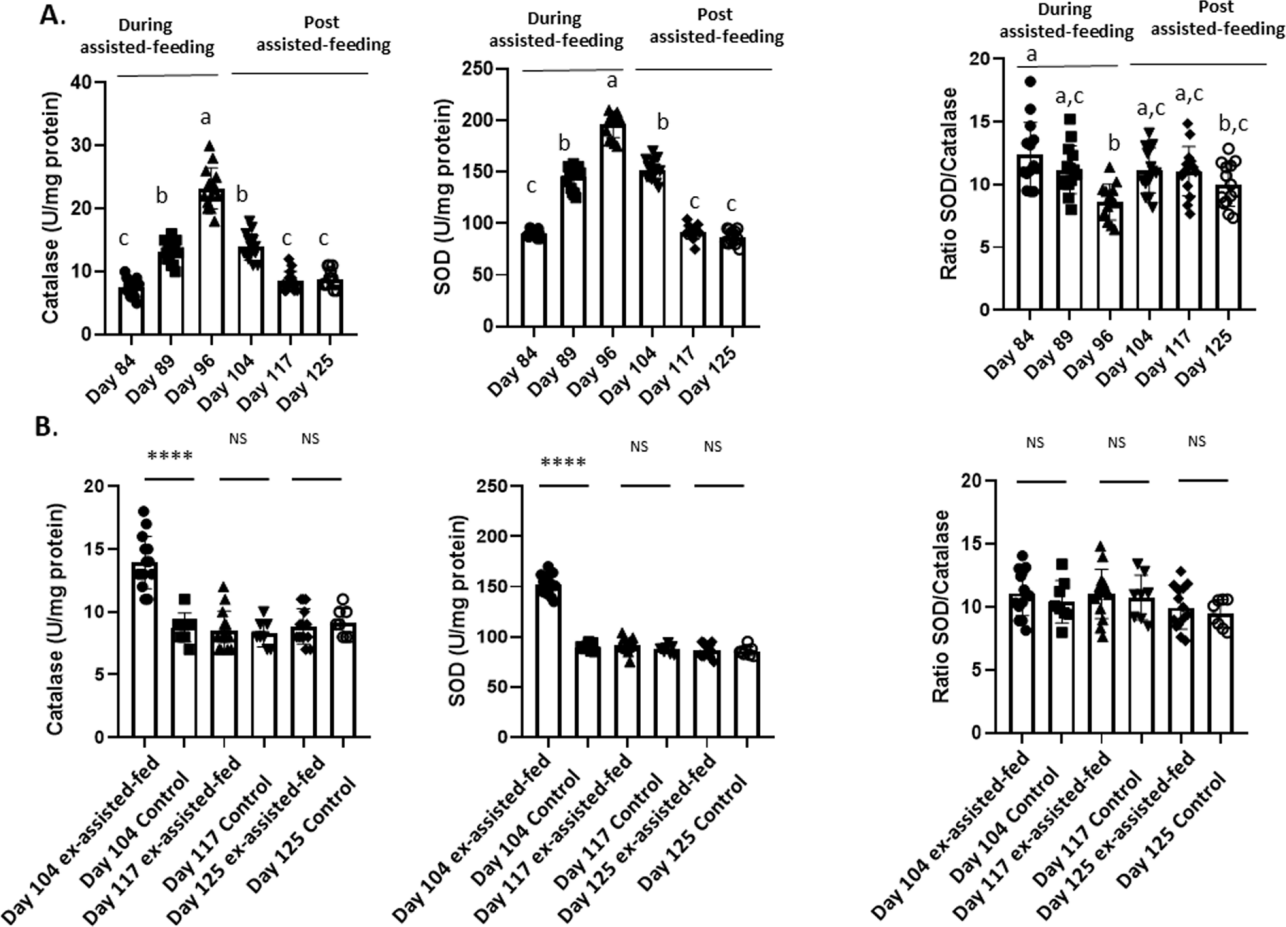
Activities of catalase and superoxide dismutase (SOD) in livers collected during and the post assisted-feeding period. **(A)** Activities of catalase and superoxide dismutase (SOD) at specific time points (n = 14) during the assisted-feeding period (days 84, 89, 96) and the post-overfeeding period (days 104, 117, 125). Values are means ± SEM. Bars with different letters (a-c) significantly differ (p < 0.05). Groups sharing the same letter are not significantly different **(B)** Comparison of activities of catalase and superoxide dismutase (SOD) at specific time points (days 104, 117, 125) between previously assisted-fed ducks (n = 14) and *ad libitum* control ducks (n = 8). Significant differences are indicated by ****p < 0.0001. NS: not significant.

**Figure 3:**
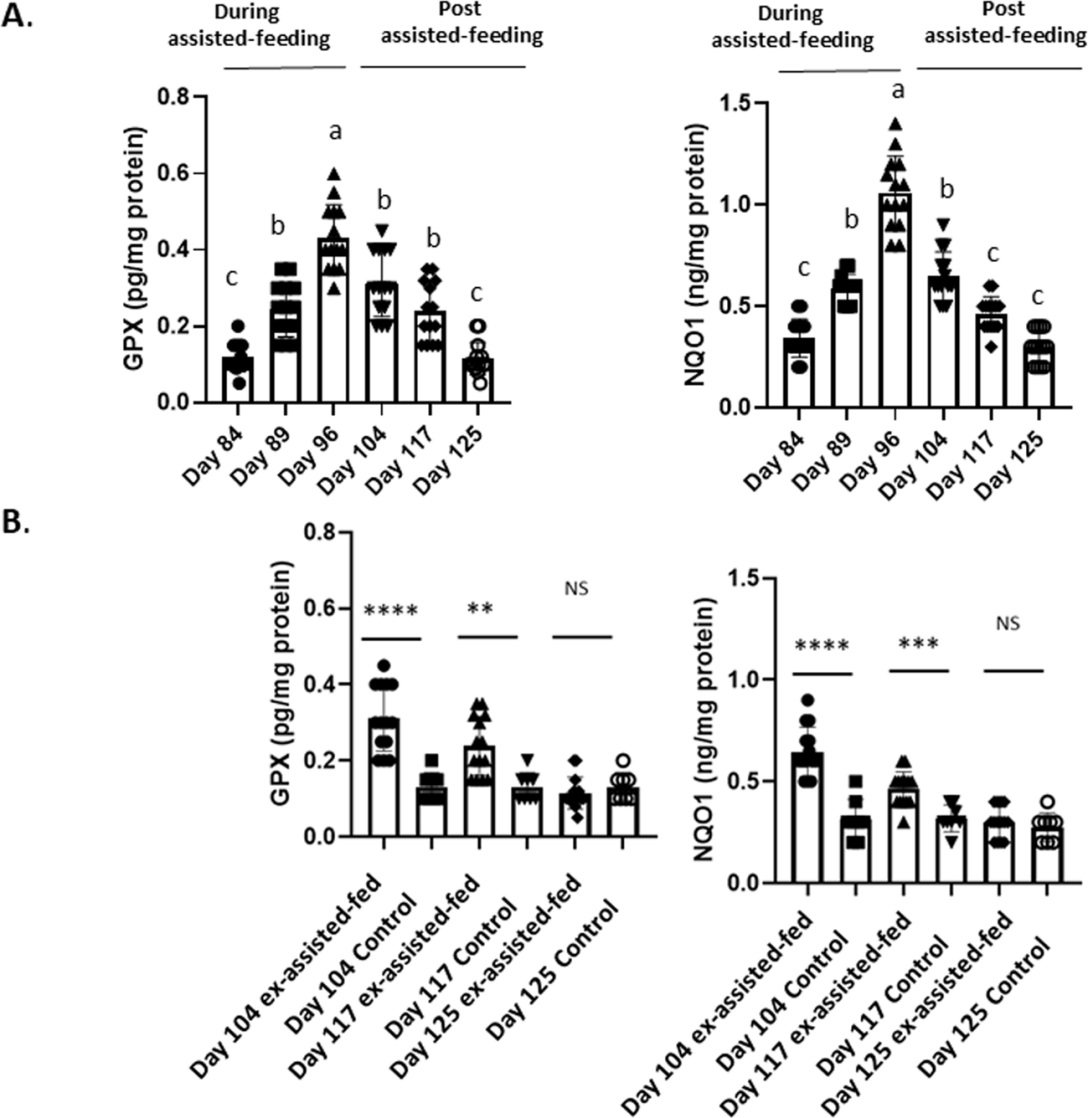
Biochemical contents of glutathione peroxidase 1 (GPX1) and nicotinamide adenine dinucleotide (phosphate) hydrogen dehydrogenase (quinone) 1 (NQO1) in livers collected during and the post assisted-feeding period. **(A)** Biochemical contents of GPX1 and NQO1 at specific time points (n = 14) during the assisted-feeding period (days 84, 89, 96) and the post-overfeeding period (days 104, 117, 125). Values are means ± SEM. Bars with different letters (a-c) significantly differ (p < 0.05). Groups sharing the same letter are not significantly different. **(B)** Comparison of at biochemical contents of GPX1 and NQO1 at specific time points (days 104, 117, 125) between previously assisted-fed ducks (n = 14) and *ad libitum* control ducks (n = 8). Significant differences are indicated by ****p < 0.0001; ***p < 0.001 and **p < 0.01. NS: not significant.

**Figure 4:**
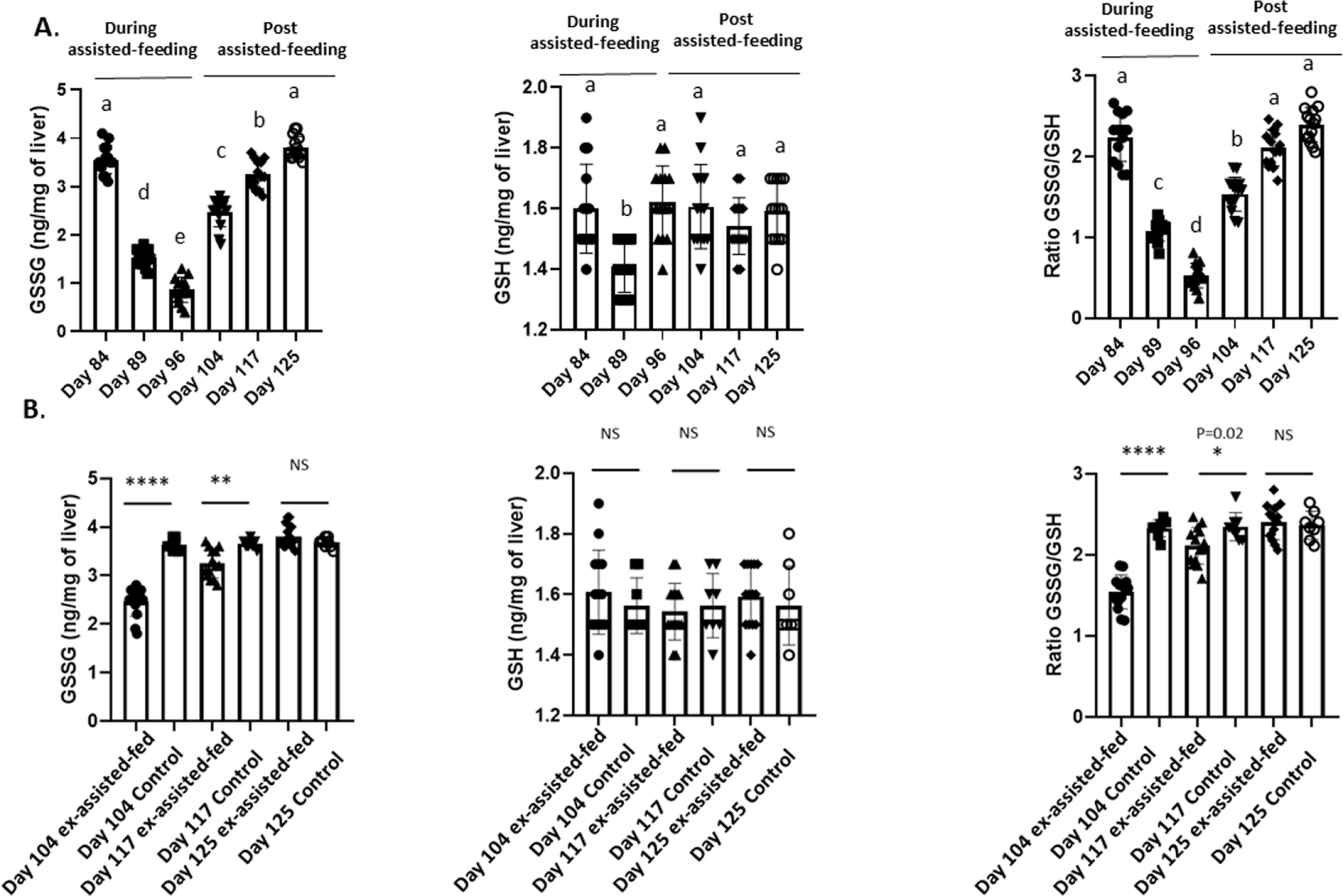
Amount of oxidized glutathione (GSSG) and reduced (GSH) in livers collected during and the post assisted-feeding period. **(A)** Values of oxidized glutathione (GSSG) and reduced (GSH) contents in liver at specific time points (n = 14) during the assisted-feeding period (days 84, 89, 96) and the post-overfeeding period (days 104, 117, 125). Values are means ± SEM. Bars with different letters (a-e) significantly differ (p < 0.05). Groups sharing the same letter are not significantly different. **(B)** Comparison of values of oxidized glutathione (GSSG) and reduced (GSH) contents in liver at specific time points (days 104, 117, 125) between previously assisted-fed ducks (n = 14) and *ad libitum* control ducks (n = 8). Significant differences are indicated by ****p < 0.0001, **p < 0.01 and *p<0.05. NS: not significant.

In the liver, SOD and Cat activities (**Figure 2**), the enzymatic antioxidant systems (GPX1 and NQO1) activities (**Figure 3**) and the content in GSSG (**Figure 4**) rapidly increased at the beginning (day 89) and all along the assisted-feeding period. However, these alterations were abolished 20 (Cat and SOD) and 29 days (for NQO1, GPX and GSSG) after the arrest of assisted-feeding. In addition, the content of GSH was not affected by the assisted-feeding (**Figures 2**–**4**).

### 3.4 Hypoxia markers in the liver of male Mule ducks during and after assisted-feeding

**Figure 5:**
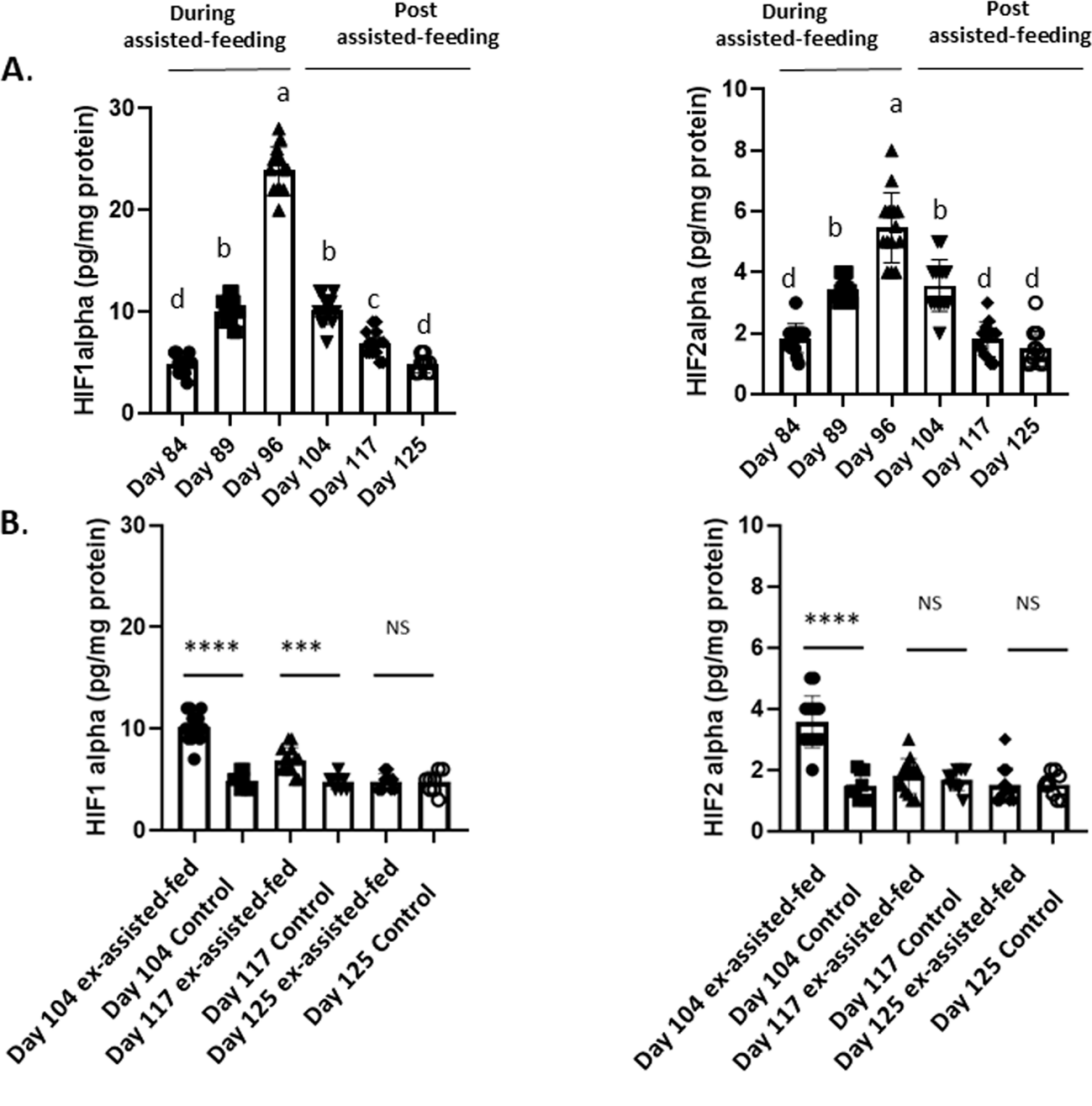
Biochemical contents of hypoxia inducible factor 1 alpha (HIF1alpha) and hypoxia inducible factor 2 alpha (HIF2alpha) in livers collected during and the post assisted-feeding period. **(A)** Biochemical contents of hypoxia inducible factor 1 alpha (HIF1alpha) and hypoxia inducible factor 2 alpha (HIF2alpha) in livers at specific time points (n = 14) during the assisted-feeding period (days 84, 89, 96) and the post-overfeeding period (days 104, 117, 125). Values are means ± SEM. Bars with different letters (a-d) significantly differ (p < 0.05). Groups sharing the same letter are not significantly different. **(B)** Comparison of hypoxia inducible factor 1 alpha (HIF1alpha) and hypoxia inducible factor 2 alpha (HIF2alpha) contents in livers at specific time points (days 104, 117, 125) between previously assisted-fed ducks (n = 14) and *ad libitum* control ducks (n = 8). Significant differences are indicated by ****p < 0.0001 and ***p < 0.001. NS: not significant.

HIF1α and HIF2α are, respectively, markers of light and severe hypoxia as reported by Rankin et al. (2009). As shown in **Figures 5A, B**, the protein expression levels of these two hypoxic factors in the liver increased similarly during the assisted-feeding period and decreased following the cessation of overfeeding. Indeed, 29 and 20 days after the arrest of assisted-feeding, the HIF-1α and HIF-2α contents were similar to those observed in the livers of control animals (**Figures 5A, B**).

### 3.5 Hepatic apoptosis in male Mule ducks during and after assisted-feeding

**Figure 6:**
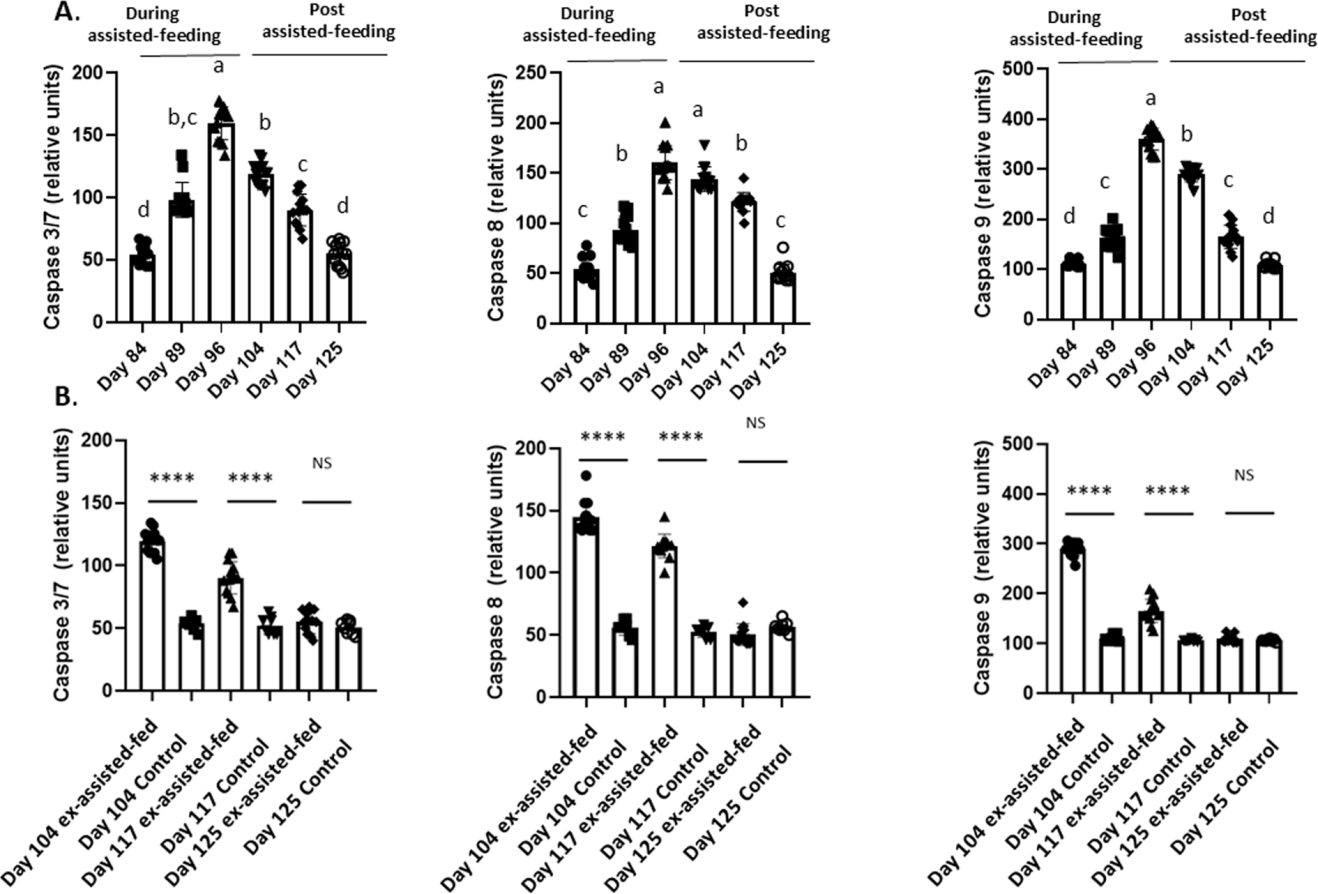
Caspase-3, caspase-8 and caspase-9 relative activities in livers collected during and the post assisted-feeding period. **(A)** Caspase-3, caspase-8 and caspase-9 relative activities in livers at specific time points (n = 14) during the assisted-feeding period (days 84, 89, 96) and the post-overfeeding period (days 104, 117, 125). Values are means ± SEM. Bars with different letters (a-d) significantly differ (p < 0.05). Groups sharing the same letter are not significantly different. **(B)** Caspase-3, caspase-8 and caspase-9 relative activities in livers at specific time points (days 104, 117, 125) between previously assisted-fed ducks (n = 14) and *ad libitum* control ducks (n = 8). Significant differences are indicated by ****p < 0.000; NS: not significant.

As shown in **Figures 6A, B**, hepatic caspase-3/7, -8, and -9 activities increased during assisted-feeding, declined following its cessation, and returned to control levels by day 29 post-assisted-feeding.

### 3.6 Gene expression analysis

To confirm the potential reversibility of alterations in lipid and cholesterol metabolism, as well as cell viability and cellular stress following assisted-feeding, the mRNA expression levels of various hepatic target genes were studied. As showed in **Table 8**, the expression levels of several genes involved in the lipogenesis (*acyl CoA synthetase long-chain 1 (acsl1), stearyl CoA desaturase (scd1), diglyceride acyl transferase 2 (dgat2)*), beta-oxidation (*acyl CoA dehydrogenase (acad) and carnitine palmitoyl transferase A (cpt1a)*), lipoprotein formation (*apolipoprotein B (apob), carnitine palmitoyl transferase A (cept1), perilipin 2 (plin2)*), fatty acid maturation and transport (*fatty acid translocase/cluster of differentiation 36 (fat/cd36) and atty acid binding protein 4 (fabp4)*), lipoprotein signaling (*low-density lipoprotein receptor (ldlr) and very low density lipoprotein receptor (vldlr)*) and cholesterol synthesis and esterification (*hydroxymethylglutaryl-CoA reductase (hmgcr) and sterol O-acyltransferase 1 (soat1)*), inflammation (*Tumor Necrosis Factor alpha (TNFα) and interleukin 8 (IL8)*) and cellular stress (*cytochrome P450 Family 2 Subfamily E Member 1 (CYP2E1) and heat shock protein beta 1 (Hspb1)*) rose steadily throughout the assisted-feeding period and declined post-cessation, reaching pre-assisted-feeding levels within 20 to 29 days. Similar results, albeit with a delayed increase during the assisted-feeding, were observed for various apoptotic markers including (*caspase-3, caspase-8, caspase-9, Bcl-2-associated X protein (Bax), B-cell lymphoma 2 (Bcl-2)*). At 29 days (day 117) or 20 days (day 125) after assisted-feeding cessation, the mRNA expression levels of all these genes were comparable to those in control livers (**Table 9**).

**Table 8:** Relative expression of genes involved in lipid metabolism, inflammation, apoptosis, and cellular stress in Mule duck livers during overfeeding (days 84–96) and after the cessation of overfeeding under *ad libitum* conditions.

	Day 84	Day 89	Day 96	Day 104	Day 117	Day 125	*P*
Lipogenesis							
*acsl1*	1.00± 0.04**^a^**	1.90± 0.11**^b^**	5.60± 0.26**^c^**	1.84± 0.23**^b^**	1.14± 0.17**^a^**	1.05± 0.22**^a^**	**0.0001**
*scd1*	1.00± 0.03**^a^**	1.80± 0.14**^b^**	2.40± 0.14**^c^**	1.76± 0.16**^b^**	1.15± 0.20**^a^**	1.06± 0.12**^a^**	**0.0001**
*dgat2*	1.00± 0.06**^a^**	1.96± 0.20**^b^**	4.97± 0.17**^c^**	1.89± 0.31**^b^**	0.98± 0.18**^a^**	1.03± 0.21**^a^**	**0.0001**
β-oxidation							
*cpt1a*	1.00± 0.12**^a^**	1.76± 0.13**^b^**	2.78± 0.11**^c^**	1.94± 0.23**^b^**	1.35± 0.20**^a^**	1.04± 0.14**^a^**	**0.0001**
*Acad*	1.00± 0.16**^a^**	2.56± 0.13**^b^**	6.57± 0.14**^c^**	2.66± 0.25**^b^**	1.31± 0.21**^a^**	1.12± 0.11**^a^**	**0.0001**
**Lipoprotein formation**							
*Cept1*	1.00± 0.05**^a^**	1.84± 0.18**^b^**	2.54± 0.11**^c^**	1.94± 0.09**^b^**	1.04± 0.18**^a^**	1.05± 0.07**^a^**	**0.0001**
*Apob*	1.00± 0.09**^a^**	2.14± 0.18**^b^**	4.15± 0.32**^c^**	2.21± 0.15**^b^**	1.08± 0.12**^a^**	1.15± 0.19**^a^**	**0.0001**
*plin2*	1.00± 0.18**^a^**	2.13± 0.12**^b^**	4.56± 0.35**^c^**	2.28± 0.23**^b^**	1.03± 0.24**^a^**	1.07± 0.11**^a^**	**0.0001**
Fatty acid maturation and transport							
*fabp4*	1.00± 0.11**^a^**	6.06± 0.34**^b^**	34.56± 2.34**^c^**	7.8± 1.42**^b^**	4.23± 0.37**^d^**	1.12± 0.11**^a^**	**0.0001**
*fat/cd36*	1.00± 0.16**^a^**	8.21± 0.21**^b^**	45.76± 3.58**^c^**	9.2± 1.56**^b^**	2.12± 0.27**^e^**	1.09± 0.31**^a^**	**0.0001**
**Lipoprotein receptors**							
*Vldr*	1.00± 0.11**^a^**	2.03± 0.08**^b^**	5.12± 0.23**^c^**	2.35± 0.23**^b^**	1.58± 0.10**^d^**	1.08± 0.10**^a^**	**0.0001**
*Ldlr*	1.00± 0.08**^a^**	1.89± 0.11**^b^**	2.95± 0.13**^c^**	1.83± 0.10**^b^**	1.24± 0.21**^a^**	1.15± 0.13**^a^**	**0.0001**
Cholesterol synthesis and esterification							
*Hmgcr*	1.00± 0.16**^a^**	1.55± 0.18**^a^**	5.55± 0.25**^b^**	1.65± 0.27**^a^**	1.24± 0.10**^a^**	1.08± 0.12**^a^**	**0.0001**
*Soat1*	1.00± 0.15**^a^**	1.62± 0.25**^a^**	4.23± 0.12**^b^**	1.58± 0.22**^a^**	1.38± 0.14**^a^**	1.10± 0.10**^a^**	**0.0001**
Inflammation							
*TNF𝛼*	1.00± 0.18**^a^**	1.22± 0.24**^a,b^**	1.66± 0.10**^b^**	1.38± 0.22**^a,b^**	1.03± 0.17**^a^**	1.05± 0.13**^a^**	**0.001**
*IL18*	1.00± 0.21**^a^**	1.65± 0.12**^b^**	2.78± 0.15**^c^**	1.8± 0.12**^b^**	1.23± 0.27**^a^**	1.08± 0.16**^a^**	**0.0001**
Apoptosis							
*Caspase-3*	1.00± 0.17**^a^**	1.28± 0.22**^a^**	5.26± 0.35**^b^**	2.0± 0.22**^b^**	1.35± 0.25**^a^**	1.02± 0.26**^a^**	**0.001**
*Caspase-8*	1.00± 0.13**^a^**	1.54± 0.17**^a^**	3.28± 0.19**^b^**	1.58± 0.18**^a^**	1.23± 0.11**^a^**	1.06± 0.17**^a^**	**0.001**
*Caspase-9*	1.00± 0.14**^a^**	1.45± 0.23**^a^**	4.55± 0.16**^b^**	1.89± 0.15**^a^**	1.27± 0.16**^a^**	1.11± 0.14**^a^**	**0.001**
*Bax*	1.00± 0.14**^a^**	1.24± 0.13**^a^**	3.38± 0.11**^b^**	1.55± 0.22**^a^**	1.13± 0.14**^a^**	1.03± 0.18**^a^**	**0.001**
*Bcl-2*	1.00± 0.11**^a^**	0.84± 0.12**^a^**	0.45± 0.07**^b^**	0.65± 0.11**^a,b^**	0.89± 0.07**^a^**	1.03± 0.09**^a^**	**0.001**
Cellular Stress							
*Cyp2e1*	1.00± 0.12**^a^**	4.21± 0.22**^b^**	7.00± 0.34**^c^**	3.94± 0.33**^b^**	2.10± 0.22**^d^**	1.10± 0.15**^a^**	**0.0001**
*Hspb1*	1.00± 0.11**^a^**	3.00± 0.12**^b^**	5.23± 0.32**^c^**	2.86± 0.22**^b^**	1.82± 0.12**^d^**	1.08± 0.22**^a^**	**0.0001**

Values are expressed as means ± SEM, n=14 at each specific time point. One-way ANOVA was performed followed by the Tukey test. Letters a,b, c and d denotes significant differences between sampling points.

The P values significant are in bold.

**Table 9:** Changes in the relative expression of genes involved in lipid metabolism, inflammation, apoptosis, and cellular stress in Mule ducks following the cessation of overfeeding, compared with control animals that were never overfed.

	Day 104		*P*	Day 117		*P*	Day 125		*P*
Lipogenesis	Ex-Overfed	Control		Ex-Overfed	Control		Ex-Overfed	Control	
*acsl1*	1.84± 0.23	1.02± 0.08	**0.020**	1.14± 0.17	1.16± 0.12	0.54	1.05± 0.22	1.10 ± 0.09	0.66
*scd1*	1.76± 0.16	1.12± 0.11	**0.030**	1.15± 0.20	1.16± 0.11	0.67	1.06± 0.12	1.11 ± 0.10	0.72
*dgat2*	1.89± 0.31	1.07± 0.05	**0.020**	0.98± 0.18	1.05± 0.15	0.71	1.03± 0.21	1.07 ± 0.11	0.75
β-oxidation									
*cpt1a*	1.94± 0.23	1.09± 0.11	**0.030**	1.35± 0.20	1.14± 0.16	0.66	1.04± 0.14	1.11 ± 0.12	0.78
*Acad*	2.66± 0.25	1.16± 0.04	**0.001**	1.31± 0.21	1.21± 0.24	0.71	1.12± 0.11	1.15 ± 0.15	0.68
Lipoprotein formation									
*Cept1*	1.94± 0.09	1.12± 0.14	**0.01**	1.04± 0.18	1.15± 0.11	0.65	1.05± 0.07	1.09 ± 0.13	0.73
*Apob*	2.21± 0.15	1.14± 0.15	**0.001**	1.08± 0.12	1.22± 0.14	0.68	1.15± 0.19	1.12± 0.14	0.78
*plin2*	2.28± 0.23	1.11± 0.12	**0.001**	1.14± 0.22	1.17± 0.23	0.71	1.07± 0.11	1.12± 0.18	0.79
Fatty acid maturation and transport									
*fabp4*	7.8± 1.42	1.23 ± 0.25	**0.001**	4.23± 0.37	1.28± 0.21	**0.001**	1.12± 0.11	1.16± 0.14	0.81
*fat/cd36*	9.2± 1.56	1.22± 0.22	**0.001**	2.12± 0.27	1.09± 0.15	**0.05**	1.09± 0.31	1.13± 0.22	0.67
Lipoprotein receptors									
*Vldr*	2.35± 0.23	1.14± 0.08	**0.001**	1.58± 0.10	1.35± 0.22	0.34	1.08± 0.10	1.17± 0.12	0.65
*Ldlr*	1.83± 0.10	1.09± 0.06	**0.01**	1.24± 0.21	1.22± 0.10	0.78	1.15± 0.13	1.25± 0.11	0.77
Cholesterol synthesis and esterification									
*Hmgcr*	1.65± 0.27	1.05± 0.16	0.07	1.24± 0.10	1.15± 0.17	0.66	1.08± 0.12	1.09± 0.10	0.65
*Soat1*	1.58± 0.22	1.22± 0.24	0.52	1.38± 0.14	1.22± 0.12	0.78	1.10± 0.10	1.15± 0.12	0.76
Inflammation									
*TNF𝛼*	1.38± 0.22	1.18± 0.22	0.57	1.03± 0.17	1.18± 0.12	0.59	1.05± 0.13	1.15± 0.11	0.78
*IL8*	1.80± 0.12	1.22± 0.08	**0.05**	1.23± 0.27	1.22± 0.15	0.67	1.08± 0.16	1.09± 0.21	0.85
Apoptosis									
*Caspase-3*	2.0± 0.22	1.12± 0.11	**0.001**	1.35± 0.25	1.21± 0.23	0.56	1.02± 0.26	1.02± 0.26	0.63
*Caspase-8*	1.58± 0.18	1.24± 0.27	0.24	1.23± 0.11	1.18± 0.15	0.67	1.06± 0.17	1.16± 0.18	0.72
*Caspase-9*	1.89± 0.15	1.15± 0.15	**0.001**	1.27± 0.16	1.15± 0.14	0.71	1.11± 0.14	1.12± 0.13	0.90
*Bax*	1.55± 0.22	1.14± 0.14	0.21	1.13± 0.14	1.15± 0.12	0.85	1.03± 0.18	1.09± 0.12	0.86
*Bcl-2*	0.65± 0.11	1.14± 0.08	**0.05**	0.89± 0.07	1.03± 0.21	0.35	1.03± 0.09	1.12± 0.10	0.76
Cellular Stress									
*Cyp2e1*	3.94± 0.33	1.11± 0.24	**0.001**	2.10± 0.22	1.04± 0.21	**0.04**	1.10± 0.15	1.12± 0.14	0.76
*Hspb1*	2.86± 0.22	1.10± 0.17	**0.001**	1.82± 0.12	1.16± 0.06	**0.01**	1.08± 0.22	1.13± 0.11	0.74

Values are expressed as means ± SEM, n=14 at each specific time point for the ex-overfed animals and n=8 for animals that have never been assisted-fed. The Ex-Overfed and control conditions were compared by a Student T Test. Letters a,b, c and d denotes significant differences between sampling points.

The values of control are relative to the value at day 84 (before the assisted-feeding).

The P values significant are in bold.

## 4 Discussion

In the present study, we investigated for the first time the potential reversibility of the hepatic changes observed after assisted-feeding in Mule duck. The present study provides a comprehensive characterization of the physiological, biochemical, and molecular changes occurring in male Mule ducks during assisted-feeding and following its cessation, with emphasis on the development and reversibility of hepatic steatosis. Our results demonstrate that assisted-feeding induces profound but largely reversible alterations in body composition, liver metabolism, oxidative stress, hypoxia, apoptosis, and gene expression, highlighting the remarkable adaptive capacity of the duck liver.

As expected, assisted-feeding induced a marked increase in body weight, liver mass, and abdominal fat, confirming the strong lipogenic drive characteristic of waterfowl under hypercaloric conditions (Hermier et al., 2003; Davail et al., 2003; Pioche et al., 2020). As already shown, the lipids accumulation is made in replace of protein and water (Rémignon and Burgues, 2023). The decrease in relative protein content observed during assisted-feeding can be attributed to the dilution effect caused by lipid accumulation during the development of hepatic steatosis. The restoration of protein levels by day 20 post-cessation highlights the liver’s structural resilience. Massive hepatic triglyceride accumulation resulting from enhanced *de novo* lipogenesis and limited very-low-density lipoprotein (VLDL) export has been described as the central mechanism underlying foie gras production (Lo et al., 2022). Following the cessation of assisted-feeding, body weight and liver mass progressively decreased and ultimately returned to levels comparable to control ducks, confirming the reversible nature of hepatic steatosis in this model. However, the persistence of elevated abdominal fat after assisted-feeding suggests slower mobilization of peripheral lipids, a phenomenon also observed in other avian models of nutritional excess (Chartrin et al., 2006). This result could also suggest a ‘memory effect’ in peripheral adipose tissue mobilization, which may consequently affect long-term metabolic health. Further investigations are required to better understand these potential metabolic alterations even if they occur outside the production cycle.

Plasma biochemical parameters further illustrate the metabolic burden imposed by assisted-feeding. Elevated cholesterol, triglycerides, and liver enzymes (ALAT, LDH, ALP) reflect hepatic lipid overload, cellular stress, and altered hepatocellular integrity (Hermier et al., 1999). The gradual normalization of these markers after assisted-feeding cessation indicates recovery of hepatic metabolic function, with differential kinetics implying distinct regulatory pathways for lipid metabolism, enzyme clearance, and bile homeostasis (Davail et al., 2003). Notably, the absence of significant changes in plasma urea suggests preserved protein metabolism and renal function throughout the experimental period, consistent with earlier reports (Zhang et al., 2005).

Assisted-feeding strongly increased liver lipid and dry matter content while decreasing relative protein levels. This shift reflects the preferential deposition of triglycerides at the expense of structural components, a hallmark of steatosis (Hermier et al., 2003). Importantly, the complete restoration of liver composition after assisted-feeding cessation indicates that these changes are dynamic and reversible. The hydroxyproline content, a validated marker of collagen and fibrosis (Rockey et al., 2015), remained unchanged during and after assisted-feeding, suggesting no detectable fibrotic remodeling. This finding aligns with studies showing that the hepatic steatosis observed in ducks remains non-fibrotic and physiologically distinct from chronic human non-alcoholic fatty liver disease (NAFLD) (Hermier et al., 2003).

The significant increases in antioxidant enzyme activities (SOD, catalase, GPX1, NQO1) and elevated GSSG levels indicate the induction of oxidative stress during assisted-feeding. Such responses have been reported in steatotic livers of both avian and mammalian origin, where enhanced lipid oxidation and mitochondrial activity elevate reactive oxygen species (ROS) production (Lo et al., 2020; Atallah et al., 2024; Baéza et al., 2013). The maintenance of GSH levels suggests that compensatory antioxidant systems were sufficiently engaged to prevent critical depletion. Hypoxia-inducible factors (HIF-1α and HIF-2α) increased during assisted-feeding, indicating intrahepatic hypoxic conditions. Hepatic hypoxia has been implicated in steatosis progression and oxidative stress in both animal models and human liver disease (Holzner and Murray, 2021). The reversal of hypoxic markers after feeding cessation underscores the dynamic interplay between metabolic load and oxygen homeostasis.

As previously shown in mule duck, assisted-feeding elicited increases in caspase-3/7, -8, and -9 activities when large quantities of lipids accumulate in the hepatic cells (Rémignon et al., 2018). This indicates activation of both intrinsic and extrinsic apoptotic pathways in response to metabolic and oxidative stress, a phenomenon reported in steatosis models (Malhi and Gores, 2008). The normalization of caspase activities following assisted-feeding cessation suggests that apoptosis is a controlled and reversible aspect of liver adaptation, rather than an irreversible path to liver failure. This controlled apoptotic modulation without significant fibrosis further differentiates physiological steatosis in ducks from chronic liver disease paradigms.

Assisted-feeding induced a coordinated transcriptional program favoring lipogenesis (*acsl1, scd1, dgat2*), fatty acid uptake and transport (*fat/cd36, fabp4*), triglyceride assembly (*apob, plin2*), and cholesterol metabolism (*hmgcr, soat1*), consistent with extensive transcriptomic and proteomic analyses in assisted-fed waterfowl (Theron et al., 2011; Rémignon et al., 2018). Concurrent upregulation of β-oxidation genes (*acad*, *cpt1a*) may reflect attempts to mitigate lipid overload, as described in adaptive responses to steatosis (Postic and Girard, 2008). Inflammatory and stress-related genes also increased during assisted-feeding, in line with ROS-mediated signaling and cytokine activation seen in steatotic tissue. The return of these gene expression patterns to baseline post-assisted-feeding highlights the flexibility of the hepatic transcriptome in response to nutritional state and metabolic burden.

## 5 Conclusion

Taken together, our results demonstrate that assisted-feeding induces profound but reversible metabolic, oxidative, hypoxic, and apoptotic responses in the liver of male Mule ducks. The rapid normalization of biochemical, and molecular parameters after assisted-feeding cessation highlights the exceptional plasticity of the duck liver and supports the use of foie gras models to study adaptive hepatic steatosis without fibrotic progression.

## Data Availability

The datasets presented in this study can be found in online repositories. The names of the repository/repositories and accession number(s) can be found in the article/**Supplementary Material**.

## Supplementary material

Supplementary Figure 1 Experimental design. Male mule ducks (n = 108) were reared at the Experimental Station for Waterfowl Breeding (INRAE Artiguères, France) as described in the Materials and Methods. At 12 weeks of age (day 84), 84 ducks were overfed for 22 meals (two meals per day for 11 days). Following the cessation of assisted-feeding, the remaining animals were fed *ad libitum*. At specific time points—during the assisted-feeding period (days 84, 89, 96) and after overfeeding (days 104, 117, 125)—14 animals were slaughtered via standardized slaughter operations (electronarcosis, bleeding, scalding, and plucking). During the assisted-feeding period, slaughter occurred two hours after the final meal. Additionally, as a control group, eight *ad libitum* fed animals (never assisted-fed) were slaughtered on days 104, 117, and 125. For all animals, blood samples were collected prior to slaughter. After dissection, the liver, breast muscle, and abdominal fat were weighed and sampled as described in the Materials and Methods.

Supplementary Figure 2 Feed intake (corn and premix without water) of mule duck during and after overfeeding.
