# Supplementary figures and images for: Dynamics and reversibility of hepatic steatosis in male Mule duck
Source: Front Physiol. 2026 Apr 16;17:1804237. doi: 10.3389/fphys.2026.1804237 (PMC13128355; doi:10.3389/fphys.2026.1804237)

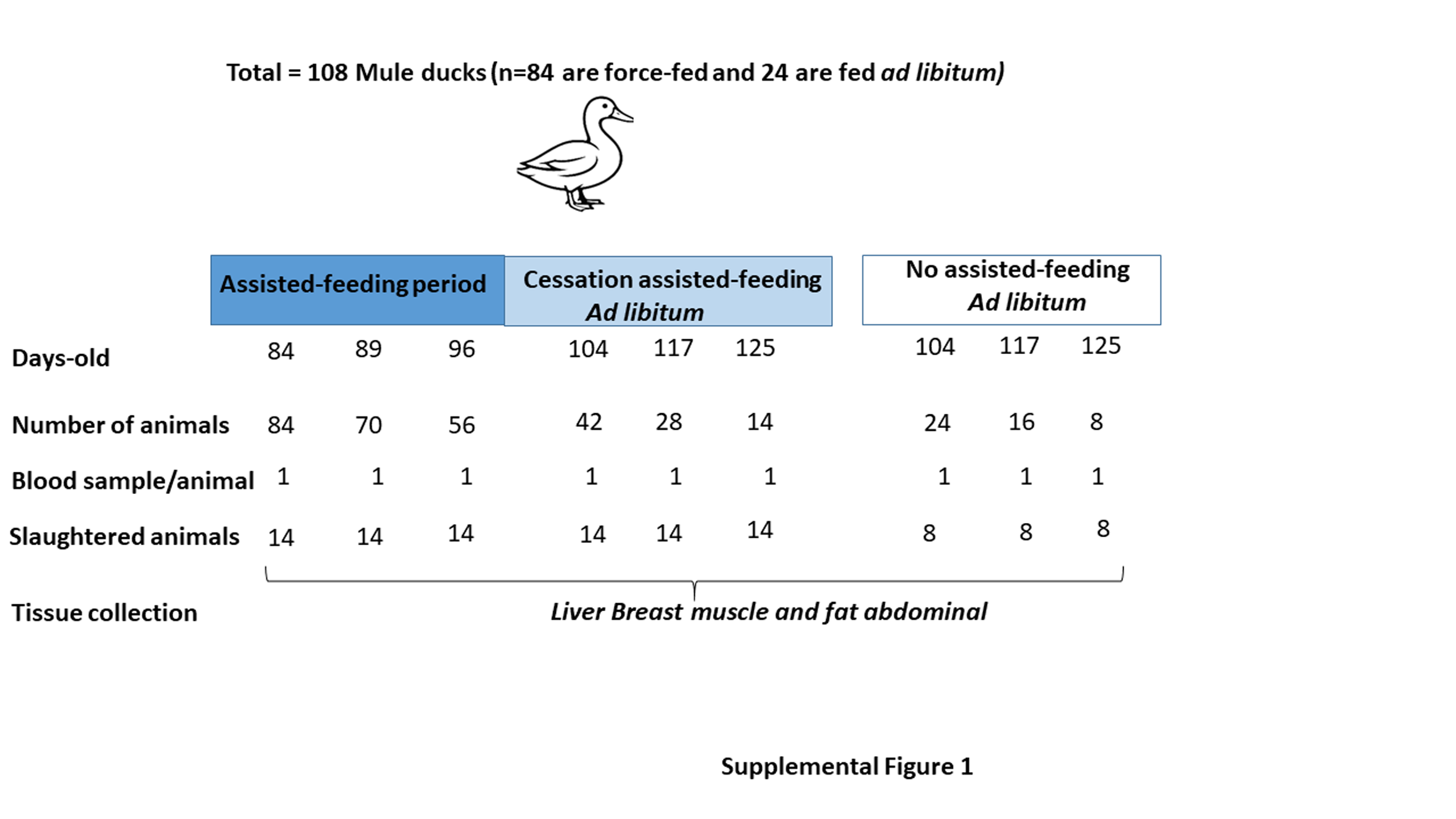

Supplement: Supplementary Figure 1 — Experimental design. Male mule ducks (n = 108) were reared at the Experimental Station for Waterfowl Breeding (INRAE Artiguères, France) as described in the Materials and Methods. At 12 weeks of age (day 84), 84 ducks were overfed for 22 meals (two meals per day for 11 days). Following the cessation of assisted-feeding, the remaining animals were fed ad libitum. At specific time points—during the assisted-feeding period (days 84, 89, 96) and after overfeeding (days 104, 117, 125)—14 animals were slaughtered via standardized slaughter operations (electronarcosis, bleeding, scalding, and plucking). During the assisted-feeding period, slaughter occurred two hours after the final meal. Additionally, as a control group, eight ad libitum fed animals (never assisted-fed) were slaughtered on days 104, 117, and 125. For all animals, blood samples were collected prior to slaughter. After dissection, the liver, breast muscle, and abdominal fat were weighed and sampled as described in the Materials and Methods. [file Image1.tif]

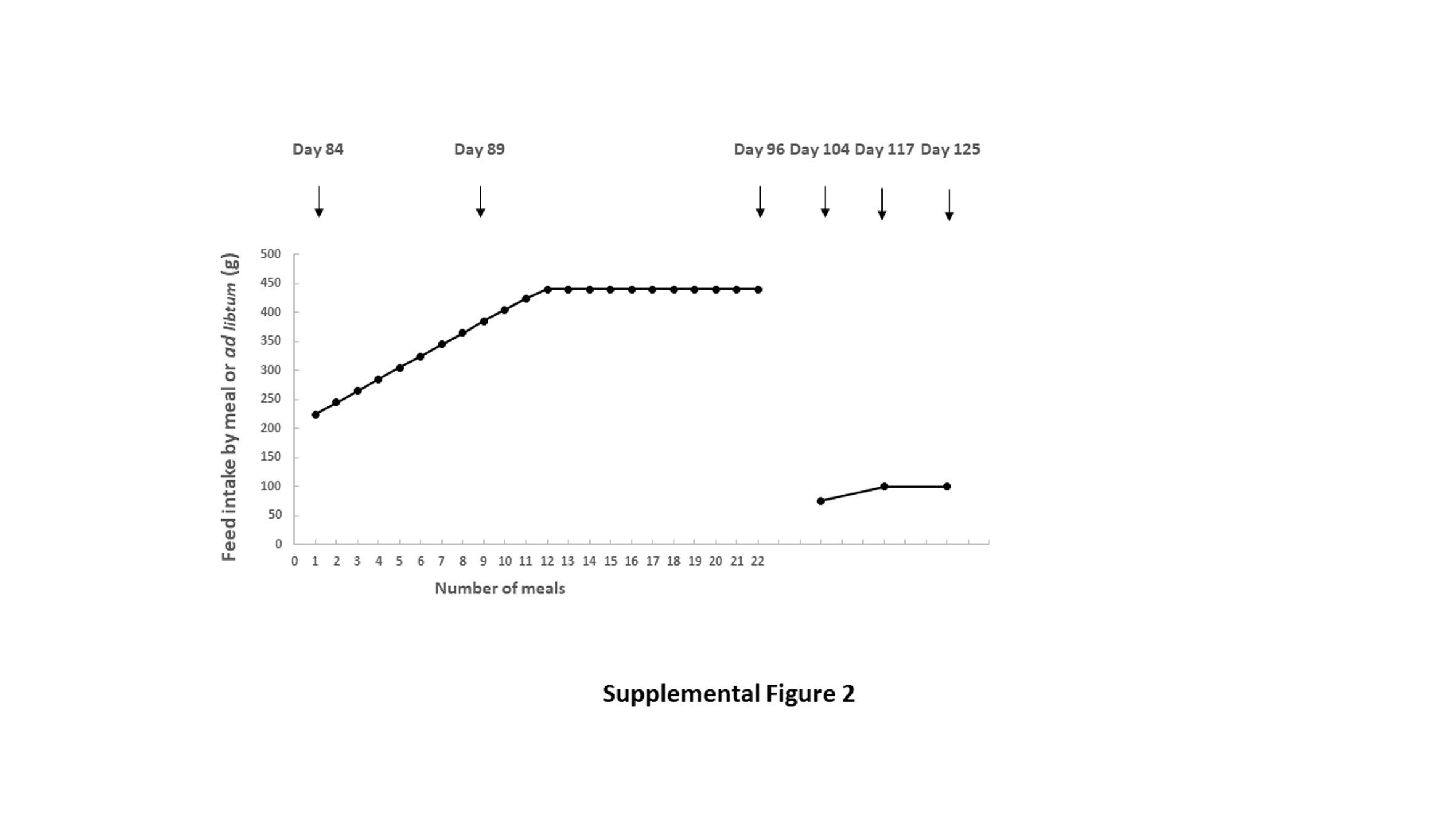

Supplement: Supplementary Figure 2 — Feed intake (corn and premix without water) of mule duck during and after overfeeding. [file Image2.tif]
