# Supplementary material for: Dynamics and reversibility of hepatic steatosis in male Mule duck
Source: Front Physiol. 2026 Apr 16;17:1804237. doi: 10.3389/fphys.2026.1804237 (PMC13128355; doi:10.3389/fphys.2026.1804237)
Supplement: Supplementary file 3 [file DataSheet1.docx]

**Supplementary materials**

**Live body weight (g):**

| Day 84 | Day 89 | Day 96 | Day 104 | Day 117 | Day 125 |
| --- | --- | --- | --- | --- | --- |
| 3883 | 5875 | 6072 | 5117 | 4690 | 5429 |
| 3494 | 4955 | 6460 | 5077 | 4927 | 4886 |
| 4250 | 4902 | 6227 | 4297 | 3819 | 3325 |
| 4159 | 5019 | 5116 | 5157 | 3859 | 4249 |
| 4354 | 4694 | 5401 | 5590 | 5290 | 5346 |
| 3886 | 4329 | 5684 | 5009 | 4098 | 5131 |
| 4001 | 4812 | 6858 | 4943 | 4108 | 3570 |
| 4234 | 4453 | 5870 | 4947 | 4676 | 5211 |
| 4366 | 5131 | 6127 | 4476 | 4702 | 5000 |
| 4058 | 4998 | 5918 | 4931 | 4147 | 4971 |
| 4423 | 4978 | 6167 | 5299 | 4715 | 5315 |
| 4753 | 4560 | 6692 | 5091 | 5563 | 4834 |
| 4812 | 5077 | 6290 | 4983 | 4933 | 5343 |
| 3201 | 4867 | 5738 | 5192 | 4014 | 5025 |
|  |  |  |  |  |  |
|  |  |  |  |  |  |
| Day 104 | Day 104 Control | Day 117 | Day 117 Control | Day 125 | Day 125 Control |
| 5117 | 3414 | 4690 | 4470 | 5429 | 4080 |
| 5077 | 4064 | 4927 | 4556 | 4886 | 3644 |
| 4297 | 4016 | 3819 | 4419 | 3325 | 4686 |
| 5157 | 3766 | 3859 | 4271 | 4249 | 4320 |
| 5590 | 4584 | 5290 | 4319 | 5346 | 4997 |
| 5009 | 3887 | 4098 | 4834 | 5131 | 4896 |
| 4943 | 3991 | 4108 | 4822 | 3570 | 4558 |
| 4947 | 4435 | 4676 | 4559 | 5211 | 4858 |
| 4476 |  | 4702 |  | 5000 |  |
| 4931 |  | 4147 |  | 4971 |  |
| 5299 |  | 4715 |  | 5315 |  |
| 5091 |  | 5563 |  | 4834 |  |
| 4983 |  | 4933 |  | 5343 |  |
| 5192 |  | 4014 |  | 5025 |  |
|  |  |  |  |  |  |

**Liver weight (g):**

| Day 84 | Day 89 | Day 96 | Day 104 | Day 117 | Day 125 |
| --- | --- | --- | --- | --- | --- |
| 46,9 | 200,8 | 869,7 | 168,24 | 60,92 | 47 |
| 39,3 | 197,56 | 762,5 | 233,27 | 58,08 | 60 |
| 42,5 | 215,83 | 550,9 | 70,72 | 63,32 | 78 |
| 55,8 | 177,8 | 374,7 | 122,01 | 149,58 | 62 |
| 51,1 | 148,5 | 674,3 | 95,64 | 60,17 | 58 |
| 53,9 | 116,44 | 746,3 | 87,12 | 46,42 | 53 |
| 39,2 | 204,91 | 590 | 115,28 | 49,09 | 50 |
| 57,3 | 224,17 | 460 | 128,85 | 62,48 | 53 |
| 57,83 | 171,6 | 594,5 | 114,48 | 46,67 | 53 |
| 59,8 | 229,5 | 457,4 | 241,8 | 71,56 | 56 |
| 67,9 | 169,5 | 637,9 | 228,85 | 57,79 | 57 |
| 78,5 | 200,9 | 476,3 | 129,87 | 77,6 | 50 |
| 65,9 | 223,5 | 610,1 | 150,8 | 71,58 | 60 |
|  | 176,4 | 599,8 | 111,27 | 58,57 | 58 |
|  |  |  |  |  |  |
|  |  |  |  |  |  |
| Day 104 | Day 104 Control | Day 117 | Day 117 Control | Day 125 | Day 125 Control |
| 168,24 | 35,12 | 60,92 | 41,12 | 47 | 57 |
| 233,27 | 42,31 | 58,08 | 49,48 | 60 | 42 |
| 70,72 | 39,1 | 63,32 | 49,67 | 78 | 43 |
| 122,01 | 41,2 | 149,58 | 40,83 | 62 | 43 |
| 95,64 | 43,36 | 60,17 | 45,13 | 58 | 55 |
| 87,12 | 43,18 | 46,42 | 49,47 | 53 | 48 |
| 115,28 | 43,59 | 49,09 | 44,99 | 50 | 65 |
| 128,85 | 51,05 | 62,48 | 38,29 | 53 | 48 |
| 114,48 |  | 46,67 |  | 53 |  |
| 241,8 |  | 71,56 |  | 56 |  |
| 228,85 |  | 57,79 |  | 57 |  |
| 129,87 |  | 77,6 |  | 50 |  |
| 150,8 |  | 71,58 |  | 60 |  |
| 111,27 |  | 58,57 |  | 58 |  |

**Breast weight (g):**

| Day 84 | Day 89 | Day 96 | Day 104 | Day 117 | Day 125 |
| --- | --- | --- | --- | --- | --- |
| 272,8 | 345,9 | 282,4 | 272,38 | 270,48 | 269 |
| 243,4 | 317,9 | 340,9 | 258,35 | 294,29 | 321 |
| 347,2 | 304 | 354,5 | 287,01 | 203,12 | 194 |
| 302,1 | 344,8 | 259,7 | 321,73 | 223,46 | 306 |
| 321,4 | 299,1 | 283,9 | 324,35 | 310,07 | 362 |
| 302,6 | 248,84 | 271,2 | 291,08 | 258,18 | 243 |
| 312,3 | 305,69 | 371,6 | 298,22 | 242,11 | 244 |
| 336,48 | 283,51 | 320,1 | 287,94 | 288,16 | 326 |
| 318,2 | 338,4 | 369,6 | 277,33 | 298,12 | 358 |
| 296,1 | 314,65 | 331,8 | 297,01 | 255,58 | 356 |
| 300,2 | 316,64 | 347,7 | 323,86 | 287,92 | 443 |
| 391,1 | 289,31 | 392,3 | 328,96 | 382,01 | 360 |
| 335,3 | 336,1 | 361,3 | 262,11 | 307,3 | 365 |
|  | 339,01 | 319,2 | 305,4 | 250,58 | 332 |
|  |  |  |  |  |  |
| Day 104 | Day 104 Control | Day 117 | Day 117 Control | Day 125 | Day 125 Control |
| 272,38 | 275,44 | 270,48 | 356,04 | 269 | 319 |
| 258,35 | 317,38 | 294,29 | 369,96 | 321 | 279 |
| 287,01 | 300,44 | 203,12 | 325,22 | 194 | 354 |
| 321,73 | 285,38 | 223,46 | 310,65 | 306 | 349 |
| 324,35 | 334,52 | 310,07 | 354,47 | 362 | 450 |
| 291,08 | 309,3 | 258,18 | 389,93 | 243 | 414 |
| 298,22 | 321,77 | 242,11 | 387,44 | 244 | 348 |
| 287,94 | 360,82 | 288,16 | 346,91 | 326 | 395 |
| 277,33 |  | 298,12 |  | 358 |  |
| 297,01 |  | 255,58 |  | 356 |  |
| 323,86 |  | 287,92 |  | 443 |  |
| 328,96 |  | 382,01 |  | 360 |  |
| 262,11 |  | 307,3 |  | 365 |  |
| 305,4 |  | 250,58 |  | 332 |  |

**Abdominal fat weight (g) :**

| Day 84 | Day 89 | Day 96 | Day 104 | Day 117 | Day 125 |
| --- | --- | --- | --- | --- | --- |
| 26,9 | 74,55 | 109,52 | 137,84 | 84,89 | 59 |
| 20,4 | 64,85 | 163,7 | 141,82 | 81,78 | 78 |
| 31 | 61,5 | 108,7 | 97,82 | 94,48 | 50 |
| 21,5 | 60,1 | 125,1 | 118,02 | 106,44 | 48 |
| 21,3 | 35,26 | 138,2 | 148,82 | 76,36 | 66 |
| 13,1 | 29,5 | 135,6 | 131,06 | 57,58 | 66 |
| 25,5 | 59,28 | 174,7 | 116,7 | 82,52 | 38 |
| 19,9 | 81,2 | 131,4 | 151,9 | 110,9 | 98 |
| 45,46 | 51,9 | 145,8 | 130,21 | 89,18 | 83 |
| 26,16 | 99,12 | 153,2 | 136,95 | 71,74 | 82 |
| 23,8 | 73,3 | 146,9 | 165,43 | 117,06 | 54 |
| 41,5 | 52,37 | 178,6 | 156,8 | 131,19 | 67 |
| 59,9 | 107,52 | 204,7 | 162,58 | 87,66 | 101 |
|  | 79,5 | 115,7 | 165,4 | 81,77 | 85 |
| Day 104 | Day 104 Control | Day 117 | Day 117 Control | Day 125 | Day 125 Control |
| 137,84 | 11,2 | 84,89 | 31,38 | 59 | 22 |
| 141,82 | 5,34 | 81,78 | 47,76 | 78 | 3 |
| 97,82 | 22,65 | 94,48 | 47,06 | 50 | 19 |
| 118,02 | 10,1 | 106,44 | 10,23 | 48 | 28 |
| 148,82 | 15,43 | 76,36 | 32,4 | 66 | 39 |
| 131,06 | 10,74 | 57,58 | 37,86 | 66 | 69 |
| 116,7 | 13,3 | 82,52 | 35,82 | 38 | 25 |
| 151,9 | 17,97 | 110,9 | 29,1 | 98 | 48 |
| 130,21 |  | 89,18 |  | 83 |  |
| 136,95 |  | 71,74 |  | 82 |  |
| 165,43 |  | 117,06 |  | 54 |  |
| 156,8 |  | 131,19 |  | 67 |  |
| 162,58 |  | 87,66 |  | 101 |  |
| 165,4 |  | 81,77 |  | 85 |  |

**Lipid % liver :**

| Day 84 | Day 89 | Day 96 | Day 104 | Day 117 | Day 125 |
| --- | --- | --- | --- | --- | --- |
| 5 | 23 | 55 | 25 | 23 | 8 |
| 4 | 25 | 60 | 26 | 22 | 5 |
| 6 | 24 | 54 | 35 | 12 | 6 |
| 3 | 26 | 65 | 34 | 11 | 4 |
| 5 | 25 | 67 | 32 | 24 | 6 |
| 5 | 28 | 54 | 28 | 14 | 7 |
| 7 | 24 | 55 | 26 | 15 | 8 |
| 5 | 25 | 59 | 28 | 22 | 4 |
| 6 | 29 | 54 | 25 | 24 | 5 |
| 7 | 24 | 53 | 24 | 15 | 6 |
| 5 | 23 | 65 | 23 | 23 | 4 |
| 6 | 25 | 50 | 27 | 22 | 4 |
| 7 | 26 | 54 | 28 | 25 | 5 |
| 7 | 24 | 52 | 26 | 24 | 6 |
|  |  |  |  |  |  |
|  |  |  |  |  |  |
| Day 104 | Day 104 Control | Day 117 | Day 117 Control | Day 125 | Day 125 Control |
| 25 | 5 | 23 | 4 | 8 | 4 |
| 26 | 5 | 22 | 5 | 5 | 6 |
| 35 | 6 | 12 | 6 | 6 | 5 |
| 34 | 8 | 11 | 8 | 4 | 4 |
| 32 | 5 | 24 | 3 | 6 | 6 |
| 28 | 4 | 14 | 4 | 7 | 4 |
| 26 | 5 | 15 | 5 | 8 | 7 |
| 28 | 3 | 22 | 4 | 4 | 4 |
| 25 |  | 24 |  | 5 |  |
| 24 |  | 15 |  | 6 |  |
| 23 |  | 23 |  | 4 |  |
| 27 |  | 22 |  | 4 |  |
| 28 |  | 25 |  | 5 |  |
| 26 |  | 24 |  | 6 |  |

**Dry matter % liver :**

| Day 84 | Day 89 | Day 96 | Day 104 | Day 117 | Day 125 |
| --- | --- | --- | --- | --- | --- |
| 34 | 44 | 72 | 56 | 40 | 35 |
| 31 | 41 | 73 | 47 | 41 | 40 |
| 35 | 45 | 78 | 41 | 35 | 33 |
| 33 | 42 | 75 | 40 | 40 | 32 |
| 34 | 41 | 74 | 45 | 36 | 31 |
| 35 | 43 | 71 | 43 | 38 | 30 |
| 33 | 46 | 78 | 42 | 34 | 34 |
| 32 | 44 | 76 | 45 | 37 | 35 |
| 36 | 42 | 79 | 56 | 36 | 32 |
| 31 | 51 | 74 | 54 | 38 | 36 |
| 30 | 47 | 73 | 62 | 32 | 30 |
| 32 | 48 | 72 | 43 | 34 | 32 |
| 33 | 43 | 70 | 42 | 36 | 33 |
|  | 44 | 82 | 62 | 38 | 32 |
|  |  |  |  |  |  |
|  |  |  |  |  |  |
| Day 104 | Day 104 Control | Day 117 | Day 117 Control | Day 125 | Day 125 Control |
| 56 | 32 | 40 | 34 | 35 | 33 |
| 47 | 34 | 41 | 36 | 40 | 31 |
| 41 | 30 | 35 | 35 | 33 | 32 |
| 40 | 31 | 40 | 33 | 32 | 35 |
| 45 | 32 | 36 | 32 | 31 | 33 |
| 43 | 33 | 38 | 35 | 30 | 34 |
| 42 | 35 | 34 | 35 | 34 | 30 |
| 45 | 36 | 37 | 34 | 35 | 32 |
| 56 |  | 36 |  | 32 |  |
| 54 |  | 38 |  | 36 |  |
| 62 |  | 32 |  | 30 |  |
| 43 |  | 34 |  | 32 |  |
| 42 |  | 36 |  | 33 |  |
| 62 |  | 38 |  | 32 |  |

**Protein % liver :**

| Day 84 | Day 89 | Day 96 | Day 104 | Day 117 | Day 125 |
| --- | --- | --- | --- | --- | --- |
| 13 | 6 | 3 | 6 | 10 | 13 |
| 14 | 5 | 4 | 4 | 11 | 14 |
| 12 | 4 | 2 | 5 | 13 | 12 |
| 15 | 3 | 3 | 6 | 12 | 14 |
| 12 | 5 | 2 | 4 | 13 | 12 |
| 10 | 4 | 4 | 3 | 14 | 10 |
| 11 | 3 | 3 | 5 | 12 | 11 |
| 13 | 6 | 3 | 4 | 11 | 14 |
| 12 | 5 | 2 | 6 | 10 | 13 |
| 14 | 4 | 3 | 7 | 14 | 12 |
| 14 | 4 | 4 | 4 | 13 | 14 |
| 12 | 5 | 2 | 5 | 12 | 14 |
| 14 | 4 | 2 | 4 | 13 | 12 |
|  | 4 | 3 | 4 | 12 | 12 |
|  |  |  |  |  |  |
|  |  |  |  |  |  |
| Day 104 | Day 104 Control | Day 117 | Day 117 Control | Day 125 | Day 125 Control |
| 56 | 32 | 40 | 34 | 35 | 33 |
| 47 | 34 | 41 | 36 | 40 | 31 |
| 41 | 30 | 35 | 35 | 33 | 32 |
| 40 | 31 | 40 | 33 | 32 | 35 |
| 45 | 32 | 36 | 32 | 31 | 33 |
| 43 | 33 | 38 | 35 | 30 | 34 |
| 42 | 35 | 34 | 35 | 34 | 30 |
| 45 | 36 | 37 | 34 | 35 | 32 |
| 56 |  | 36 |  | 32 |  |
| 54 |  | 38 |  | 36 |  |
| 62 |  | 32 |  | 30 |  |
| 43 |  | 34 |  | 32 |  |
| 42 |  | 36 |  | 33 |  |
| 62 |  | 38 |  | 32 |  |

**OH-Pro liver :**

| Day 84 | Day 89 | Day 96 | Day 104 | Day 117 | Day 125 |
| --- | --- | --- | --- | --- | --- |
| 0,46285714 | 0,45240385 |  | 0,38653846 | 0,49607843 | 0,48 |
| 0,39658742 | 0,43113208 | 0,42884615 | 0,48 | 0,33557692 | 0,47941176 |
| 0,38786408 | 0,4575 | 0,35970874 | 0,43823529 | 0,37735849 | 0,458972 |
| 0,37376238 | 0,34902913 | 0,35490196 | 0,36897452 |  | 0,49411765 |
| 0,56407767 | 0,56 | 0,37745098 | 0,36442308 | 0,45678921 | 0,46897125 |
| 0,37058824 | 0,531 | 0,44571429 | 0,32697812 | 0,47892157 | 0,43698217 |
| 0,42673267 | 0,45339806 | 0,35952381 | 0,32156863 | 0,4615 | 0,49428571 |
| 0,42727273 | 0,46730769 | 0,33490566 | 0,39320388 | 0,44059406 | 0,42772277 |
| 0,52722772 | 0,46067961 |  | 0,39752475 | 0,4730198 | 0,47029703 |
| 0,41099057 | 0,42425743 | 0,45769231 | 0,34934783 | 0,42378641 |  |
| 0,44166667 | 0,37663551 |  | 0,34779412 | 0,4052381 | 0,47920792 |
|  | 0,49215686 | 0,37631579 | 0,35294118 | 0,47358491 | 0,46116505 |
|  | 0,54455446 | 0,41095238 | 0,37663551 | 0,42216981 | 0,46911765 |
|  | 0,44660194 | 0,35825243 | 0,38269231 | 0,38823529 | 0,48173077 |
|  |  |  |  |  |  |
|  |  |  |  |  |  |
| Day 104 | Day 104 Control | Day 117 | Day 117 Control | Day 125 | Day 125 Control |
| 0,38653846 | 0,46058824 | 0,49607843 |  | 0,48 | 0,43564356 |
| 0,48 | 0,49782355 | 0,33557692 |  | 0,47941176 | 0,46078431 |
| 0,43823529 |  | 0,37735849 |  | 0,458972 | 0,46273585 |
| 0,36897452 | 0,44068627 |  |  | 0,49411765 | 0,526 |
| 0,36442308 | 0,49117647 | 0,45678921 |  | 0,46897125 | 0,5315534 |
| 0,32697812 | 0,44029126 | 0,47892157 |  | 0,43698217 | 0,48268 |
| 0,32156863 | 0,48142857 | 0,4615 |  | 0,49428571 | 0,42087379 |
| 0,39320388 | 0,46519608 | 0,44059406 |  | 0,42772277 | 0,48137255 |
| 0,39752475 |  | 0,4730198 |  | 0,47029703 |  |
| 0,34934783 |  | 0,42378641 |  |  |  |
| 0,34779412 |  | 0,4052381 |  | 0,47920792 |  |
| 0,35294118 |  | 0,47358491 |  | 0,46116505 |  |
| 0,37663551 |  | 0,42216981 |  | 0,46911765 |  |
| 0,38269231 |  | 0,38823529 |  | 0,48173077 |  |

**Cholesterol plasma**

| Day 84 | Day 89 | Day 96 | Day 104 | Day 117 | Day 125 |
| --- | --- | --- | --- | --- | --- |
| 4,5 | 4,6 | 5,54 | 5,01 | 4,15 | 4,13 |
| 3,5 | 5,1 | 6,35 | 4,12 | 4,2 | 4,12 |
| 4 | 4,5 | 5,34 | 4,56 | 3,78 | 3,98 |
| 4,3 | 4,45 | 5,35 | 4,1 | 3,56 | 4,29 |
| 4,5 | 5,15 | 4,67 | 3,85 | 3,45 | 4,06 |
| 4,95 | 4,98 | 5,86 | 4,13 | 4,56 | 4,13 |
| 5,13 | 4,98 | 6,1 | 4,89 | 4,15 | 4,97 |
| 4,78 | 5,12 | 4,24 | 4,15 | 4,13 | 4,14 |
| 3,98 | 5,23 | 6,56 | 4,98 | 4,17 | 4,25 |
| 4,67 | 4,56 | 5,89 | 4,13 | 4,23 | 4,23 |
| 4,35 | 4,78 | 4,12 | 4,98 | 3,98 | 4,34 |
| 4,78 | 4,98 | 6,01 | 4,1 | 3,67 | 4,31 |
| 4,5 | 4,12 | 5 | 4,56 | 4,85 | 4,25 |
| 4,4 | 4,4 | 5,1 | 4,34 | 4,56 | 4,18 |
| Day 104 | Day 104 Control | Day 117 | Day 117 Control | Day 125 | Day 125 Control |
| 5,01 | 4,14 | 4,15 | 4,2 | 4,13 | 4,13 |
| 4,12 | 4,87 | 4,2 | 3,76 | 4,12 | 4,12 |
| 4,56 | 3,56 | 3,78 | 4,12 | 3,98 | 3,98 |
| 4,1 | 4,15 | 3,56 | 4,13 | 4,29 | 4,29 |
| 3,85 | 4,05 | 3,45 | 4,35 | 4,06 | 4,06 |
| 4,13 | 4,78 | 4,56 | 4,89 | 4,13 | 4,13 |
| 4,89 | 4,67 | 4,15 | 3,99 | 4,97 | 4,97 |
| 4,15 | 4,25 | 4,13 | 4,12 | 4,14 | 4,14 |
| 4,98 |  | 4,17 |  | 4,25 |  |
| 4,13 |  | 4,23 |  | 4,23 |  |
| 4,98 |  | 3,98 |  | 4,34 |  |
| 4,1 |  | 3,67 |  | 4,31 |  |
| 4,56 |  | 4,85 |  | 4,25 |  |
| 4,34 |  | 4,56 |  | 4,18 |  |

**ALAT Plasma :**

| Day 84 | Day 89 | Day 96 | Day 104 | Day 117 | Day 125 |
| --- | --- | --- | --- | --- | --- |
| 23,2 | 28,5 | 44,6 | 27,6 | 25,4 | 22 |
| 24 | 27,8 | 45,3 | 26,7 | 24,3 | 24 |
| 22,3 | 30,4 | 43,4 | 26,5 | 24,7 | 24 |
| 21,5 | 32,4 | 42,5 | 26,7 | 24,5 | 24 |
| 24,5 | 35,4 | 45,6 | 30,4 | 23,3 | 25 |
| 25,2 | 32,4 | 43,2 | 32,5 | 23,4 | 23 |
| 22,3 | 30,4 | 42,5 | 31,3 | 23,5 | 22 |
| 21,3 | 27,4 | 41,5 | 30,2 | 24,6 | 21,8 |
| 22,4 | 26,7 | 38,9 | 25,6 | 23,7 | 22,5 |
| 24,5 | 28,6 | 48,5 | 30,4 | 24,7 | 22,8 |
| 23,4 | 33,2 | 42,3 | 28,5 | 24,7 | 22,9 |
| 25,6 | 33,4 | 41,5 | 28,5 | 23,5 | 23,6 |
| 24,5 | 32,4 | 40,6 | 26,5 | 23,7 | 23,8 |
| 22,4 | 32,5 | 45,6 | 25,6 | 23,6 | 23,9 |

| Day 104 | Day 104 Control | Day 117 | Day 117 Control | Day 125 | Day 125 Control |
| --- | --- | --- | --- | --- | --- |
| 27,6 | 22,2 | 25,4 | 22,3 | 22 | 22,4 |
| 26,7 | 22,1 | 24,3 | 23,1 | 24 | 22,2 |
| 26,5 | 23,1 | 24,7 | 22,4 | 24 | 22,5 |
| 26,7 | 22,5 | 24,5 | 23,1 | 24 | 23,1 |
| 30,4 | 23,6 | 23,3 | 22,6 | 25 | 21,8 |
| 32,5 | 21,9 | 23,4 | 23,1 | 23 | 22,1 |
| 31,3 | 22 | 23,5 | 22,5 | 22 | 23,1 |
| 30,2 | 23,2 | 24,6 | 21,8 | 21,8 | 22,8 |
| 25,6 |  | 23,7 |  | 22,5 |  |
| 30,4 |  | 24,7 |  | 22,8 |  |
| 28,5 |  | 24,7 |  | 22,9 |  |
| 28,5 |  | 23,5 |  | 23,6 |  |
| 26,5 |  | 23,7 |  | 23,8 |  |
| 25,6 |  | 23,6 |  | 23,9 |  |

**LDH Plasma :**

| Day 84 | Day 89 | Day 96 | Day 104 | Day 117 | Day 125 |
| --- | --- | --- | --- | --- | --- |
| 3012 | 3500 | 5234 | 3756 | 3241 | 3012 |
| 3045 | 3546 | 4789 | 4234 | 3023 | 3056 |
| 3023 | 3578 | 4891 | 4123 | 3140 | 3012 |
| 3012 | 3600 | 4567 | 4567 | 2875 | 3012 |
| 2900 | 3756 | 4789 | 3785 | 3012 | 2875 |
| 3134 | 3045 | 4698 | 3567 | 3045 | 3124 |
| 3045 | 3875 | 4784 | 3672 | 2986 | 3022 |
| 3012 | 3750 | 4856 | 3451 | 3045 | 3000 |
| 3010 | 3045 | 4956 | 3567 | 3067 | 3008 |
| 2875 | 3786 | 4923 | 3674 | 3214 | 2984 |
| 3145 | 3902 | 4023 | 3781 | 3087 | 3140 |
| 3140 | 4034 | 4934 | 4001 | 3145 | 3145 |
| 3100 | 3567 | 4956 | 4012 | 2906 | 3189 |
| 2909 | 3785 | 4812 | 3867 | 3049 | 3102 |

| Day 104 | Day 104 Control | Day 117 | Day 117 Control | Day 125 | Day 125 Control |
| --- | --- | --- | --- | --- | --- |
| 3756 | 3023 | 3241 | 3002 | 3012 | 2986 |
| 4234 | 3045 | 3023 | 3012 | 3056 | 3024 |
| 4123 | 2867 | 3140 | 2987 | 3012 | 3045 |
| 4567 | 2965 | 2875 | 3005 | 3012 | 2986 |
| 3785 | 3102 | 3012 | 3045 | 2875 | 3089 |
| 3567 | 3012 | 3045 | 3012 | 3124 | 3024 |
| 3672 | 3049 | 2986 | 2897 | 3022 | 2987 |
| 3451 | 3040 | 3045 | 2900 | 3000 | 3012 |
| 3567 |  | 3067 |  | 3008 |  |
| 3674 |  | 3214 |  | 2984 |  |
| 3781 |  | 3087 |  | 3140 |  |
| 4001 |  | 3145 |  | 3145 |  |
| 4012 |  | 2906 |  | 3189 |  |
| 3867 |  | 3049 |  | 3102 |  |

**ALP Plasma :**

| Day 84 | Day 89 | Day 96 | Day 104 | Day 117 | Day 125 |
| --- | --- | --- | --- | --- | --- |
| 150 | 143 | 132 | 139 | 154 | 152 |
| 143 | 144 | 146 | 142 | 143 | 143 |
| 135 | 135 | 128 | 141 | 145 | 145 |
| 133 | 145 | 125 | 139 | 152 | 148 |
| 145 | 143 | 124 | 142 | 148 | 135 |
| 147 | 135 | 132 | 145 | 138 | 158 |
| 139 | 138 | 129 | 123 | 138 | 143 |
| 140 | 140 | 117 | 148 | 143 | 159 |
| 142 | 142 | 128 | 150 | 138 | 140 |
| 138 | 138 | 135 | 126 | 139 | 138 |
| 144 | 137 | 129 | 128 | 135 | 139 |
| 135 | 135 | 132 | 123 | 132 | 155 |
| 153 | 142 | 125 | 127 | 131 | 157 |
| 155 | 140 | 130 | 143 | 136 | 153 |
| Day 104 | Day 104 Control | Day 117 | Day 117 Control | Day 125 | Day 125 Control |
| 139 | 143 | 154 | 142 | 152 | 142 |
| 142 | 150 | 143 | 153 | 143 | 135 |
| 141 | 134 | 145 | 135 | 145 | 155 |
| 139 | 155 | 152 | 148 | 148 | 136 |
| 142 | 132 | 148 | 138 | 135 | 135 |
| 145 | 142 | 138 | 141 | 158 | 138 |
| 123 | 140 | 138 | 140 | 143 | 144 |
| 148 | 142 | 143 | 142 | 159 | 150 |
| 150 |  | 138 |  | 140 |  |
| 126 |  | 139 |  | 138 |  |
| 128 |  | 135 |  | 139 |  |
| 123 |  | 132 |  | 155 |  |
| 127 |  | 131 |  | 157 |  |
| 143 |  | 136 |  | 153 |  |

**Bilirubin Plasma :**

| Day 84 | Day 89 | Day 96 | Day 104 | Day 117 | Day 125 |
| --- | --- | --- | --- | --- | --- |
| 5 | 8 | 15 | 6 | 5 | 5 |
| 6 | 7 | 16 | 8 | 4 | 4 |
| 4 | 9 | 17 | 9 | 5 | 4 |
| 3 | 8 | 15 | 7 | 4 | 5 |
| 5 | 9 | 14 | 8 | 3 | 3 |
| 6 | 8 | 15 | 7 | 4 | 4 |
| 4 | 7 | 16 | 6 | 5 | 5 |
| 5 | 9 | 14 | 7 | 3 | 4 |
| 6 | 7 | 13 | 8 | 3 | 5 |
| 5 | 10 | 15 | 9 | 4 | 3 |
| 6 | 9 | 16 | 12 | 3 | 4 |
| 4 | 9 | 14 | 9 | 4 | 4 |
| 4 | 8 | 15 | 8 | 5 | 3 |
| 3 | 7 | 13 | 9 | 4 | 4 |

| Day 104 | Day 104 Control | Day 117 | Day 117 Control | Day 125 | Day 125 Control |
| --- | --- | --- | --- | --- | --- |
| 6 | 5 | 5 | 4 | 5 | 5 |
| 8 | 4 | 4 | 5 | 4 | 4 |
| 9 | 5 | 5 | 6 | 4 | 6 |
| 7 | 3 | 4 | 3 | 5 | 4 |
| 8 | 4 | 3 | 4 | 3 | 3 |
| 7 | 5 | 4 | 5 | 4 | 4 |
| 6 | 6 | 5 | 3 | 5 | 5 |
| 7 | 3 | 3 | 4 | 3 | 3 |
| 8 |  | 3 |  | 5 |  |
| 9 |  | 4 |  | 3 |  |
| 12 |  | 3 |  | 4 |  |
| 9 |  | 4 |  | 4 |  |
| 8 |  | 5 |  | 3 |  |
| 9 |  | 4 |  | 4 |  |

**Triglycerid Plasma :**

| Day 84 | Day 89 | Day 96 | Day 104 | Day 117 | Day 125 |
| --- | --- | --- | --- | --- | --- |
| 1,1 | 2,5 | 4,5 | 3,1 | 1,6 | 1,5 |
| 1,3 | 2,3 | 4,3 | 2,7 | 2,2 | 2 |
| 0,9 | 2,4 | 3,9 | 2,5 | 1,9 | 1,2 |
| 1,3 | 2,4 | 4,3 | 2 | 1,3 | 0,8 |
| 1,1 | 2,1 | 4,2 | 2,4 | 1,2 | 1,1 |
| 1,2 | 2,7 | 3,8 | 1,9 | 1,1 | 1,3 |
| 1,2 | 2,7 | 3,9 | 2,3 | 0,9 | 1,2 |
| 1,1 | 2,5 | 4,1 | 2,5 | 1,2 | 1,1 |
| 1,1 | 2,7 | 4 | 2,7 | 1,4 | 1,3 |
| 1 | 2,8 | 3,8 | 2,6 | 1,3 | 1 |
| 1,1 | 2,8 | 3,7 | 2,5 | 1,3 | 1,2 |
| 1,2 | 2,5 | 4,1 | 2,8 | 1,2 | 1,3 |
| 0,9 | 2,8 | 4 | 2,7 | 1,2 | 1,2 |
| 0,8 | 2,9 | 3,9 | 2,5 | 1,1 | 1,1 |

| Day 104 | Day 104 Control | Day 117 | Day 117 Control | Day 125 | Day 125 Control |
| --- | --- | --- | --- | --- | --- |
| 3,1 | 0,9 | 1,6 | 1,1 | 1,5 | 1 |
| 2,7 | 0,8 | 2,2 | 1,2 | 2 | 0,9 |
| 2,5 | 1,2 | 1,9 | 1 | 1,2 | 1,1 |
| 2 | 1,1 | 1,3 | 0,9 | 0,8 | 1,1 |
| 2,4 | 1,1 | 1,2 | 1,2 | 1,1 | 0,9 |
| 1,9 | 0,8 | 1,1 | 1 | 1,3 | 1,2 |
| 2,3 | 0,9 | 0,9 | 0,9 | 1,2 | 0,9 |
| 2,5 | 1,2 | 1,2 | 1,1 | 1,1 | 1,1 |
| 2,7 |  | 1,4 |  | 1,3 |  |
| 2,6 |  | 1,3 |  | 1 |  |
| 2,5 |  | 1,3 |  | 1,2 |  |
| 2,8 |  | 1,2 |  | 1,3 |  |
| 2,7 |  | 1,2 |  | 1,2 |  |
| 2,5 |  | 1,1 |  | 1,1 |  |

**Creatinin Plasma :**

| Day 84 | Day 89 | Day 96 | Day 104 | Day 117 | Day 125 |
| --- | --- | --- | --- | --- | --- |
| 11,3 | 14,4 | 25,6 | 15,6 | 11,6 | 11,5 |
| 11,5 | 14,6 | 22,4 | 15,8 | 11,6 | 11,4 |
| 11,5 | 15,7 | 23,6 | 16,9 | 11,6 | 11,4 |
| 11,7 | 16,7 | 26,6 | 16,7 | 11,7 | 11,5 |
| 18,4 | 12,3 | 26,5 | 15,9 | 12,1 | 12,8 |
| 15,3 | 12,6 | 25,4 | 14,7 | 12,6 | 11,9 |
| 8,9 | 13,7 | 28,4 | 13,9 | 14,5 | 14,3 |
| 10,6 | 12,4 | 22,3 | 18,4 | 12,3 | 11,4 |
| 11,4 | 10,5 | 17,5 | 20,4 | 11,8 | 11,6 |
| 11,4 | 11,8 | 15,6 | 15,6 | 11,6 | 11,2 |
| 14,3 | 14,5 | 16,7 | 13,4 | 11,5 | 11,4 |
| 9,3 | 16,7 | 19,3 | 14,5 | 10,4 | 12,2 |
| 10,3 | 18,9 | 28,5 | 13,2 | 10,5 | 12,1 |
| 14,5 | 19,2 | 25,4 | 13,5 | 12,3 | 12,6 |

| Day 104 | Day 104 Control | Day 117 | Day 117 Control | Day 125 | Day 125 Control |
| --- | --- | --- | --- | --- | --- |
| 15,6 | 11,4 | 11,6 | 11,6 | 11,5 | 11,6 |
| 15,8 | 11,5 | 11,6 | 11,8 | 11,4 | 11,4 |
| 16,9 | 12,4 | 11,6 | 12,4 | 11,4 | 12,1 |
| 16,7 | 11,8 | 11,7 | 11,5 | 11,5 | 11,9 |
| 15,9 | 13,8 | 12,1 | 11,9 | 12,8 | 12,6 |
| 14,7 | 11,5 | 12,6 | 12,7 | 11,9 | 12,3 |
| 13,9 | 11,7 | 14,5 | 11,6 | 14,3 | 11,8 |
| 18,4 | 11,9 | 12,3 | 11,8 | 11,4 | 11,6 |
| 20,4 |  | 11,8 |  | 11,6 |  |
| 15,6 |  | 11,6 |  | 11,2 |  |
| 13,4 |  | 11,5 |  | 11,4 |  |
| 14,5 |  | 10,4 |  | 12,2 |  |
| 13,2 |  | 10,5 |  | 12,1 |  |
| 13,5 |  | 12,3 |  | 12,6 |  |

**Urea Plasma :**

| Day 84 | Day 89 | Day 96 | Day 104 | Day 117 | Day 125 |
| --- | --- | --- | --- | --- | --- |
| 0,62 | 0,65 | 0,68 | 0,62 | 0,68 | 0,61 |
| 0,65 | 0,64 | 0,61 | 0,61 | 0,67 | 0,63 |
| 0,5 | 0,72 | 0,52 | 0,65 | 0,65 | 0,54 |
| 0,67 | 0,64 | 0,65 | 0,83 | 0,69 | 0,67 |
| 0,58 | 0,57 | 0,56 | 0,56 | 0,73 | 0,59 |
| 0,56 | 0,55 | 0,55 | 0,54 | 0,6 | 0,57 |
| 0,6 | 0,67 | 0,62 | 0,58 | 0,62 | 0,58 |
| 0,61 | 0,63 | 0,82 | 0,65 | 0,63 | 0,6 |
| 0,63 | 0,56 | 0,74 | 0,66 | 0,64 | 0,65 |
| 0,65 | 0,58 | 0,76 | 0,65 | 0,73 | 0,66 |
| 0,59 | 0,6 | 0,72 | 0,67 | 0,58 | 0,67 |
| 0,58 | 0,56 | 0,68 | 0,64 | 0,54 | 0,59 |
| 0,6 | 0,61 | 0,67 | 0,65 | 0,67 | 0,62 |
| 0,59 | 0,58 | 0,62 | 0,66 | 0,62 | 0,61 |

| Day 104 | Day 104 Control | Day 117 | Day 117 Control | Day 125 | Day 125 Control |
| --- | --- | --- | --- | --- | --- |
| 0,62 | 0,61 | 0,68 | 0,68 | 0,61 | 0,6 |
| 0,61 | 0,62 | 0,67 | 0,65 | 0,63 | 0,62 |
| 0,65 | 0,63 | 0,65 | 0,64 | 0,54 | 0,56 |
| 0,83 | 0,67 | 0,69 | 0,65 | 0,67 | 0,65 |
| 0,56 | 0,57 | 0,73 | 0,67 | 0,59 | 0,6 |
| 0,54 | 0,56 | 0,6 | 0,62 | 0,57 | 0,55 |
| 0,58 | 0,57 | 0,62 | 0,61 | 0,58 | 0,56 |
| 0,65 | 0,62 | 0,63 | 0,6 | 0,6 | 0,58 |
| 0,66 |  | 0,64 |  | 0,65 |  |
| 0,65 |  | 0,73 |  | 0,66 |  |
| 0,67 |  | 0,58 |  | 0,67 |  |
| 0,64 |  | 0,54 |  | 0,59 |  |
| 0,65 |  | 0,67 |  | 0,62 |  |
| 0,66 |  | 0,62 |  | 0,61 |  |

**Catalase**

| Day 84 | Day 89 | Day 96 | Day 104 | Day 117 | Day 125 |
| --- | --- | --- | --- | --- | --- |
| 8 | 15 | 22 | 13 | 8 | 11 |
| 7 | 14 | 21 | 15 | 10 | 8 |
| 6 | 12 | 20 | 14 | 11 | 10 |
| 7 | 11 | 24 | 11 | 7 | 11 |
| 9 | 10 | 22 | 13 | 12 | 7 |
| 8 | 13 | 18 | 12 | 7 | 8 |
| 6 | 16 | 22 | 13 | 8 | 9 |
| 8 | 15 | 24 | 13 | 8 | 8 |
| 5 | 14 | 23 | 14 | 8 | 7 |
| 8 | 13 | 28 | 11 | 8 | 8 |
| 7 | 12 | 26 | 16 | 9 | 9 |
| 10 | 11 | 25 | 17 | 7 | 9 |
| 9 | 15 | 30 | 18 | 9 | 11 |
| 8 | 14 | 20 | 15 | 7 | 8 |

| Day 104 | Day 104 Control | Day 117 | Day 117 Control | Day 125 | Day 125 Control |
| --- | --- | --- | --- | --- | --- |
| 13 | 8 | 8 | 8 | 11 | 10 |
| 15 | 9 | 10 | 7 | 8 | 8 |
| 14 | 9 | 11 | 8 | 10 | 9 |
| 11 | 7 | 7 | 9 | 11 | 8 |
| 13 | 11 | 12 | 8 | 7 | 11 |
| 12 | 8 | 7 | 7 | 8 | 8 |
| 13 | 9 | 8 | 10 | 9 | 9 |
| 13 | 9 | 8 | 9 | 8 | 10 |
| 14 |  | 8 |  | 7 |  |
| 11 |  | 8 |  | 8 |  |
| 16 |  | 9 |  | 9 |  |
| 17 |  | 7 |  | 9 |  |
| 18 |  | 9 |  | 11 |  |
| 15 |  | 7 |  | 8 |  |

**SOD**

| Day 84 | Day 89 | Day 96 | Day 104 | Day 117 | Day 125 |
| --- | --- | --- | --- | --- | --- |
| 90 | 150 | 200 | 162 | 95 | 81 |
| 95 | 125 | 202 | 151 | 90 | 91 |
| 88 | 134 | 178 | 145 | 92 | 85 |
| 92 | 141 | 180 | 155 | 85 | 84 |
| 85 | 152 | 210 | 170 | 92 | 75 |
| 92 | 130 | 205 | 153 | 98 | 80 |
| 96 | 128 | 208 | 148 | 88 | 81 |
| 89 | 155 | 205 | 135 | 91 | 95 |
| 91 | 151 | 190 | 155 | 93 | 90 |
| 88 | 149 | 180 | 145 | 91 | 92 |
| 93 | 148 | 201 | 143 | 89 | 85 |
| 95 | 152 | 175 | 139 | 75 | 82 |
| 85 | 155 | 202 | 161 | 99 | 95 |
| 87 | 158 | 204 | 164 | 104 | 94 |

| Day 104 | Day 104 Control | Day 117 | Day 117 Control | Day 125 | Day 125 Control |
| --- | --- | --- | --- | --- | --- |
| 162 | 90 | 95 | 89 | 81 | 87 |
| 151 | 89 | 90 | 90 | 91 | 81 |
| 145 | 91 | 92 | 88 | 85 | 95 |
| 155 | 94 | 85 | 82 | 84 | 85 |
| 170 | 88 | 92 | 88 | 75 | 88 |
| 153 | 95 | 98 | 94 | 80 | 85 |
| 148 | 85 | 88 | 85 | 81 | 82 |
| 135 | 86 | 91 | 83 | 95 | 83 |
| 155 |  | 93 |  | 90 |  |
| 145 |  | 91 |  | 92 |  |
| 143 |  | 89 |  | 85 |  |
| 139 |  | 75 |  | 82 |  |
| 161 |  | 99 |  | 95 |  |
| 164 |  | 104 |  | 94 |  |

**GPX**

| Day 84 | Day 89 | Day 96 | Day 104 | Day 117 | Day 125 |
| --- | --- | --- | --- | --- | --- |
| 0,1 | 0,2 | 0,4 | 0,2 | 0,2 | 0,15 |
| 0,2 | 0,3 | 0,5 | 0,3 | 0,15 | 0,1 |
| 0,15 | 0,15 | 0,35 | 0,25 | 0,2 | 0,2 |
| 0,09 | 0,15 | 0,45 | 0,3 | 0,25 | 0,1 |
| 0,1 | 0,25 | 0,35 | 0,25 | 0,15 | 0,09 |
| 0,15 | 0,2 | 0,4 | 0,3 | 0,2 | 0,12 |
| 0,12 | 0,3 | 0,3 | 0,2 | 0,15 | 0,05 |
| 0,05 | 0,25 | 0,6 | 0,4 | 0,32 | 0,08 |
| 0,1 | 0,35 | 0,5 | 0,4 | 0,35 | 0,1 |
| 0,15 | 0,15 | 0,4 | 0,3 | 0,25 | 0,1 |
| 0,1 | 0,3 | 0,5 | 0,4 | 0,35 | 0,12 |
| 0,1 | 0,35 | 0,4 | 0,4 | 0,3 | 0,1 |
| 0,15 | 0,25 | 0,55 | 0,45 | 0,32 | 0,2 |
| 0,1 | 0,2 | 0,35 | 0,2 | 0,15 | 0,1 |

| Day 104 | Day 104 NG | Day 117 | Day 117 NG | Day 125 | Day 125 NG |
| --- | --- | --- | --- | --- | --- |
| 0,2 | 0,1 | 0,2 | 0,15 | 0,15 | 0,2 |
| 0,3 | 0,15 | 0,15 | 0,1 | 0,1 | 0,15 |
| 0,25 | 0,2 | 0,2 | 0,1 | 0,2 | 0,1 |
| 0,3 | 0,15 | 0,25 | 0,15 | 0,1 | 0,1 |
| 0,25 | 0,1 | 0,15 | 0,1 | 0,09 | 0,1 |
| 0,3 | 0,1 | 0,2 | 0,1 | 0,12 | 0,15 |
| 0,2 | 0,15 | 0,15 | 0,2 | 0,05 | 0,15 |
| 0,4 | 0,1 | 0,32 | 0,15 | 0,08 | 0,1 |
| 0,4 |  | 0,35 |  | 0,1 |  |
| 0,3 |  | 0,25 |  | 0,1 |  |
| 0,4 |  | 0,35 |  | 0,12 |  |
| 0,4 |  | 0,3 |  | 0,1 |  |
| 0,45 |  | 0,32 |  | 0,2 |  |
| 0,2 |  | 0,15 |  | 0,1 |  |

**NQO1**

| Day 84 | Day 89 | Day 96 | Day 104 | Day 117 | Day 125 |
| --- | --- | --- | --- | --- | --- |
| 0,3 | 0,6 | 1 | 0,8 | 0,5 | 0,3 |
| 0,4 | 0,5 | 1,15 | 0,9 | 0,4 | 0,2 |
| 0,5 | 0,65 | 1,1 | 0,6 | 0,5 | 0,4 |
| 0,3 | 0,7 | 0,9 | 0,7 | 0,6 | 0,4 |
| 0,2 | 0,6 | 0,8 | 0,65 | 0,4 | 0,3 |
| 0,3 | 0,6 | 1,2 | 0,6 | 0,5 | 0,3 |
| 0,5 | 0,7 | 1,1 | 0,5 | 0,4 | 0,2 |
| 0,4 | 0,5 | 1 | 0,7 | 0,3 | 0,3 |
| 0,3 | 0,6 | 1,4 | 0,6 | 0,6 | 0,4 |
| 0,3 | 0,6 | 1,3 | 0,8 | 0,5 | 0,3 |
| 0,2 | 0,5 | 1,2 | 0,6 | 0,4 | 0,2 |
| 0,3 | 0,5 | 0,9 | 0,5 | 0,5 | 0,2 |
| 0,4 | 0,6 | 0,8 | 0,6 | 0,5 | 0,3 |
| 0,4 | 0,6 | 1 | 0,5 | 0,4 | 0,4 |

| Day 84 | Day 89 | Day 96 | Day 104 | Day 117 | Day 125 | Day 104 NG | Day 117 NG | Day 125 NG |
| --- | --- | --- | --- | --- | --- | --- | --- | --- |
| 0,3 | 0,6 | 1 | 0,8 | 0,5 | 0,3 | 0,3 | 0,3 | 0,3 |
| 0,4 | 0,5 | 1,15 | 0,9 | 0,4 | 0,2 | 0,4 | 0,4 | 0,3 |
| 0,5 | 0,65 | 1,1 | 0,6 | 0,5 | 0,4 | 0,5 | 0,35 | 0,2 |
| 0,3 | 0,7 | 0,9 | 0,7 | 0,6 | 0,4 | 0,3 | 0,3 | 0,4 |
| 0,2 | 0,6 | 0,8 | 0,65 | 0,4 | 0,3 | 0,3 | 0,2 | 0,3 |
| 0,3 | 0,6 | 1,2 | 0,6 | 0,5 | 0,3 | 0,2 | 0,4 | 0,2 |
| 0,5 | 0,7 | 1,1 | 0,5 | 0,4 | 0,2 | 0,2 | 0,3 | 0,3 |
| 0,4 | 0,5 | 1 | 0,7 | 0,3 | 0,3 | 0,3 | 0,3 | 0,2 |
| 0,3 | 0,6 | 1,4 | 0,6 | 0,6 | 0,4 |  |  |  |
| 0,3 | 0,6 | 1,3 | 0,8 | 0,5 | 0,3 |  |  |  |
| 0,2 | 0,5 | 1,2 | 0,6 | 0,4 | 0,2 |  |  |  |
| 0,3 | 0,5 | 0,9 | 0,5 | 0,5 | 0,2 |  |  |  |
| 0,4 | 0,6 | 0,8 | 0,6 | 0,5 | 0,3 |  |  |  |
| 0,4 | 0,6 | 1 | 0,5 | 0,4 | 0,4 |  |  |  |

**GSSG**

| Day 84 | Day 89 | Day 96 | Day 104 | Day 117 | Day 125 |
| --- | --- | --- | --- | --- | --- |
| 3,5 | 1,3 | 0,8 | 1,8 | 2,9 | 3,6 |
| 3,6 | 1,5 | 1 | 2,2 | 3 | 3,6 |
| 3,2 | 1,6 | 1,2 | 2,5 | 3,1 | 3,7 |
| 4 | 1,4 | 1,3 | 2,6 | 2,8 | 3,9 |
| 3,1 | 1,5 | 1,1 | 1,9 | 3,2 | 3,7 |
| 3,5 | 1,7 | 0,5 | 2,7 | 3,5 | 4,2 |
| 3,2 | 1,8 | 1 | 2,6 | 3,6 | 4 |
| 3,5 | 1,4 | 0,7 | 2,5 | 3,7 | 3,8 |
| 3,6 | 1,2 | 0,4 | 2,8 | 3,6 | 3,7 |
| 3,8 | 1,7 | 0,6 | 2,6 | 2,9 | 4,2 |
| 4,1 | 1,5 | 0,8 | 2,7 | 3,2 | 4,1 |
| 3,8 | 1,6 | 0,9 | 2,5 | 3,3 | 3,6 |
| 3,5 | 1,4 | 1 | 2,7 | 3,5 | 3,7 |
| 3,4 | 1,7 | 0,8 | 2,4 | 3,1 | 3,5 |

| Day 104 | Day 104 Control | Day 117 | Day 117 Control | Day 125 | Day 125 Control |
| --- | --- | --- | --- | --- | --- |
| 1,8 | 3,7 | 2,9 | 3,6 | 3,6 | 3,8 |
| 2,2 | 3,8 | 3 | 3,5 | 3,6 | 3,5 |
| 2,5 | 3,5 | 3,1 | 3,7 | 3,7 | 3,7 |
| 2,6 | 3,5 | 2,8 | 3,6 | 3,9 | 3,7 |
| 1,9 | 3,6 | 3,2 | 3,7 | 3,7 | 3,6 |
| 2,7 | 3,8 | 3,5 | 3,7 | 4,2 | 3,6 |
| 2,6 | 3,6 | 3,6 | 3,8 | 4 | 3,7 |
| 2,5 | 3,5 | 3,7 | 3,6 | 3,8 | 3,8 |
| 2,8 |  | 3,6 |  | 3,7 |  |
| 2,6 |  | 2,9 |  | 4,2 |  |
| 2,7 |  | 3,2 |  | 4,1 |  |
| 2,5 |  | 3,3 |  | 3,6 |  |
| 2,7 |  | 3,5 |  | 3,7 |  |
| 2,4 |  | 3,1 |  | 3,5 |  |

**GSH**

| Day 84 | Day 89 | Day 96 | Day 104 | Day 117 | Day 125 |
| --- | --- | --- | --- | --- | --- |
| 1,5 | 1,3 | 1,6 | 1,5 | 1,5 | 1,4 |
| 1,4 | 1,3 | 1,5 | 1,5 | 1,4 | 1,6 |
| 1,8 | 1,4 | 1,7 | 1,5 | 1,5 | 1,7 |
| 1,5 | 1,5 | 1,6 | 1,4 | 1,5 | 1,5 |
| 1,6 | 1,3 | 1,6 | 1,6 | 1,4 | 1,5 |
| 1,5 | 1,5 | 1,4 | 1,7 | 1,6 | 1,5 |
| 1,8 | 1,4 | 1,7 | 1,6 | 1,5 | 1,6 |
| 1,5 | 1,4 | 1,5 | 1,5 | 1,5 | 1,7 |
| 1,9 | 1,5 | 1,6 | 1,5 | 1,6 | 1,5 |
| 1,5 | 1,4 | 1,5 | 1,6 | 1,7 | 1,6 |
| 1,6 | 1,3 | 1,7 | 1,7 | 1,7 | 1,7 |
| 1,7 | 1,5 | 1,8 | 1,7 | 1,6 | 1,7 |
| 1,5 | 1,4 | 1,7 | 1,9 | 1,5 | 1,6 |
| 1,6 | 1,5 | 1,8 | 1,8 | 1,6 | 1,7 |

| Day 104 | Day 104 Control | Day 117 | Day 117 Control | Day 125 | Day 125 Control |
| --- | --- | --- | --- | --- | --- |
| 1,5 | 1,5 | 1,5 | 1,6 | 1,4 | 1,5 |
| 1,5 | 1,7 | 1,4 | 1,5 | 1,6 | 1,5 |
| 1,5 | 1,5 | 1,5 | 1,7 | 1,7 | 1,7 |
| 1,4 | 1,5 | 1,5 | 1,5 | 1,5 | 1,4 |
| 1,6 | 1,5 | 1,4 | 1,6 | 1,5 | 1,5 |
| 1,7 | 1,6 | 1,6 | 1,7 | 1,5 | 1,5 |
| 1,6 | 1,7 | 1,5 | 1,4 | 1,6 | 1,6 |
| 1,5 | 1,5 | 1,5 | 1,5 | 1,7 | 1,8 |
| 1,5 |  | 1,6 |  | 1,5 |  |
| 1,6 |  | 1,7 |  | 1,6 |  |
| 1,7 |  | 1,7 |  | 1,7 |  |
| 1,7 |  | 1,6 |  | 1,7 |  |
| 1,9 |  | 1,5 |  | 1,6 |  |
| 1,8 |  | 1,6 |  | 1,7 |  |

**HIF1**

| Day 84 | Day 89 | Day 96 | Day 104 | Day 117 | Day 125 |
| --- | --- | --- | --- | --- | --- |
| 5 | 9 | 22 | 11 | 7 | 5 |
| 4 | 10 | 25 | 12 | 6 | 6 |
| 6 | 11 | 28 | 10 | 5 | 4 |
| 4 | 8 | 27 | 9 | 8 | 5 |
| 3 | 10 | 24 | 10 | 7 | 6 |
| 5 | 12 | 22 | 11 | 9 | 4 |
| 6 | 8 | 25 | 10 | 6 | 4 |
| 4 | 9 | 22 | 12 | 5 | 5 |
| 5 | 10 | 24 | 12 | 7 | 4 |
| 6 | 11 | 20 | 9 | 6 | 4 |
| 5 | 12 | 26 | 7 | 6 | 5 |
| 5 | 9 | 24 | 10 | 7 | 4 |
| 4 | 10 | 25 | 9 | 8 | 6 |
| 5 | 11 | 22 | 11 | 9 | 5 |

| Day 104 | Day 104 Control | Day 117 | Day 117 Control | Day 125 | Day 125 Control |
| --- | --- | --- | --- | --- | --- |
| 4 | 1,5 | 1,3 | 1,5 | 1 | 2 |
| 3 | 1 | 2 | 1 | 1,5 | 1,5 |
| 4 | 2,1 | 1,5 | 2 | 1 | 1,6 |
| 3 | 1,3 | 2 | 1,4 | 2 | 2 |
| 4 | 1 | 1,2 | 2 | 3 | 1,8 |
| 3 | 1,3 | 2 | 1,5 | 2 | 1,5 |
| 4 | 1 | 2,4 | 1,8 | 1 | 1,2 |
| 3 | 2 | 1 | 2 | 1 | 1 |
| 2 | 2 | 1,6 | 1,8 | 1,5 | 1 |
| 5 |  | 3 |  | 1,3 |  |
| 3 |  | 2,2 |  | 1,4 |  |
| 4 |  | 2 |  | 1 |  |
| 3 |  | 1 |  | 2 |  |
| 5 |  | 2 |  | 1,2 |  |

**HIF2**

| Day 84 | Day 89 | Day 96 | Day 104 | Day 117 | Day 125 |
| --- | --- | --- | --- | --- | --- |
| 2 | 4 | 5 | 4 | 1,3 | 1 |
| 2 | 3 | 6 | 3 | 2 | 1,5 |
| 1,2 | 3,5 | 4 | 4 | 1,5 | 1 |
| 2 | 3,5 | 5 | 3 | 2 | 2 |
| 1,5 | 3 | 4 | 4 | 1,2 | 3 |
| 2 | 4 | 5,5 | 3 | 2 | 2 |
| 2 | 3,6 | 6 | 4 | 2,4 | 1 |
| 1,6 | 4 | 4 | 3 | 1 | 1 |
| 2 | 3,2 | 5 | 2 | 1,6 | 1,5 |
| 3 | 3,4 | 6 | 5 | 3 | 1,3 |
| 2 | 3,5 | 7 | 3 | 2,2 | 1,4 |
| 1 | 3,4 | 8 | 4 | 2 | 1 |
| 1,5 | 3,2 | 5 | 3 | 1 | 2 |
| 2 | 3 | 6 | 5 | 2 | 1,2 |

| Day 104 | Day 104 Control | Day 117 | Day 117 Control | Day 125 | Day 125 Control |
| --- | --- | --- | --- | --- | --- |
| 4 | 1,5 | 1,3 | 1,5 | 1 | 2 |
| 3 | 1 | 2 | 1 | 1,5 | 1,5 |
| 4 | 2,1 | 1,5 | 2 | 1 | 1,6 |
| 3 | 1,3 | 2 | 1,4 | 2 | 2 |
| 4 | 1 | 1,2 | 2 | 3 | 1,8 |
| 3 | 1,3 | 2 | 1,5 | 2 | 1,5 |
| 4 | 1 | 2,4 | 1,8 | 1 | 1,2 |
| 3 | 2 | 1 | 2 | 1 | 1 |
| 2 | 2 | 1,6 | 1,8 | 1,5 | 1 |
| 5 |  | 3 |  | 1,3 |  |
| 3 |  | 2,2 |  | 1,4 |  |
| 4 |  | 2 |  | 1 |  |
| 3 |  | 1 |  | 2 |  |
| 5 |  | 2 |  | 1,2 |  |

**Casp3/7**

| Day 84 | Day 89 | Day 96 | Day 104 | Day 117 | Day 125 |
| --- | --- | --- | --- | --- | --- |
| 50 | 95 | 145 | 110 | 90 | 55 |
| 65 | 90 | 160 | 125 | 89 | 50 |
| 55 | 100 | 165 | 132 | 110 | 45 |
| 60 | 125 | 170 | 125 | 110 | 40 |
| 55 | 90 | 156 | 119 | 90 | 43 |
| 45 | 100 | 167 | 112 | 105 | 55 |
| 60 | 90 | 173 | 120 | 90 | 62 |
| 55 | 95 | 134 | 110 | 95 | 45 |
| 50 | 100 | 145 | 105 | 98 | 56 |
| 45 | 90 | 167 | 123 | 88 | 64 |
| 46 | 134 | 143 | 110 | 67 | 65 |
| 57 | 89 | 167 | 120 | 78 | 56 |
| 67 | 89 | 167 | 123 | 80 | 67 |
| 50 | 90 | 178 | 134 | 74 | 65 |

| Day 104 | Day 104 Control | Day 117 | Day 117 Control | Day 125 | Day 125 Control |
| --- | --- | --- | --- | --- | --- |
| 110 | 53 | 90 | 45 | 55 | 50 |
| 125 | 55 | 89 | 45 | 50 | 56 |
| 132 | 56 | 110 | 56 | 45 | 54 |
| 125 | 60 | 110 | 63 | 40 | 45 |
| 119 | 54 | 90 | 45 | 43 | 43 |
| 112 | 45 | 105 | 48 | 55 | 46 |
| 120 | 56 | 90 | 59 | 62 | 57 |
| 110 | 49 | 95 | 56 | 45 | 54 |
| 105 |  | 98 |  | 56 |  |
| 123 |  | 88 |  | 64 |  |
| 110 |  | 67 |  | 65 |  |
| 120 |  | 78 |  | 56 |  |
| 123 |  | 80 |  | 67 |  |
| 134 |  | 74 |  | 65 |  |

**Casp8**

| Day 84 | Day 89 | Day 96 | Day 104 | Day 117 | Day 125 |
| --- | --- | --- | --- | --- | --- |
| 56 | 94 | 178 | 156 | 112 | 76 |
| 67 | 85 | 178 | 146 | 123 | 54 |
| 56 | 100 | 145 | 134 | 100 | 56 |
| 55 | 86 | 201 | 178 | 145 | 57 |
| 45 | 115 | 176 | 156 | 123 | 43 |
| 68 | 85 | 145 | 134 | 119 | 47 |
| 78 | 76 | 134 | 135 | 122 | 46 |
| 49 | 110 | 155 | 136 | 121 | 46 |
| 45 | 78 | 157 | 143 | 123 | 45 |
| 50 | 84 | 158 | 142 | 125 | 43 |
| 53 | 107 | 155 | 145 | 121 | 52 |
| 48 | 117 | 154 | 134 | 118 | 48 |
| 48 | 88 | 156 | 138 | 125 | 46 |
| 39 | 85 | 158 | 145 | 124 | 47 |

| Day 104 | Day 104 Control | Day 117 | Day 117 Control | Day 125 | Day 125 Control |
| --- | --- | --- | --- | --- | --- |
| 156 | 49 | 112 | 54 | 76 | 57 |
| 146 | 56 | 123 | 56 | 54 | 58 |
| 134 | 63 | 100 | 46 | 56 | 59 |
| 178 | 56 | 145 | 54 | 57 | 54 |
| 156 | 57 | 123 | 53 | 43 | 55 |
| 134 | 56 | 119 | 52 | 47 | 65 |
| 135 | 63 | 122 | 58 | 46 | 54 |
| 136 | 46 | 121 | 46 | 46 | 50 |
| 143 |  | 123 |  | 45 |  |
| 142 |  | 125 |  | 43 |  |
| 145 |  | 121 |  | 52 |  |
| 134 |  | 118 |  | 48 |  |
| 138 |  | 125 |  | 46 |  |
| 145 |  | 124 |  | 47 |  |

**Casp9**

| Day 84 | Day 89 | Day 96 | Day 104 | Day 117 | Day 125 |
| --- | --- | --- | --- | --- | --- |
| 112 | 150 | 375 | 302 | 150 | 110 |
| 110 | 176 | 380 | 256 | 200 | 110 |
| 124 | 180 | 356 | 274 | 157 | 123 |
| 123 | 156 | 367 | 302 | 134 | 124 |
| 110 | 179 | 374 | 298 | 167 | 105 |
| 109 | 178 | 389 | 302 | 167 | 105 |
| 108 | 145 | 345 | 289 | 125 | 108 |
| 107 | 175 | 387 | 302 | 189 | 109 |
| 105 | 145 | 356 | 306 | 209 | 111 |
| 110 | 167 | 370 | 289 | 167 | 106 |
| 112 | 202 | 325 | 276 | 157 | 105 |
| 105 | 124 | 330 | 289 | 156 | 104 |
| 107 | 145 | 367 | 302 | 178 | 102 |
| 109 | 152 | 325 | 278 | 156 | 102 |

| Day 104 | Day 104 Control | Day 117 | Day 117 Control | Day 125 | Day 125 Control |
| --- | --- | --- | --- | --- | --- |
| 302 | 110 | 150 | 110 | 110 | 110 |
| 256 | 109 | 200 | 105 | 110 | 108 |
| 274 | 120 | 157 | 106 | 123 | 106 |
| 302 | 105 | 134 | 104 | 124 | 105 |
| 298 | 120 | 167 | 105 | 105 | 106 |
| 302 | 105 | 167 | 107 | 105 | 108 |
| 289 | 104 | 125 | 105 | 108 | 105 |
| 302 | 105 | 189 | 103 | 109 | 102 |
| 306 |  | 209 |  | 111 |  |
| 289 |  | 167 |  | 106 |  |
| 276 |  | 157 |  | 105 |  |
| 289 |  | 156 |  | 104 |  |
| 302 |  | 178 |  | 102 |  |
| 278 |  | 156 |  | 102 |  |
